# Cycloastragenol Attenuates Angiotensin II-Induced Cardiac Remodeling in Association with Enhanced EGFR Lysosomal Turnover and Changes in MAPK Signaling and Autophagy

**DOI:** 10.3390/ph19091360

**Published:** 2026-08-27

**Authors:** Dongsheng Wei, Han Li, Mei Zhao, Mai Liu, Yongyue Liu, Menglan Zhao, Huiming Cao, Xiaoqing Zhang

**Affiliations:** 1 School of Life Sciences, Beijing University of Chinese Medicine, Beijing 100029, China; xfyw2586@163.com (D.W.);; 2 Key Laboratory of Ministry of Education for TCM Viscera-State Theory and Applications, Liaoning University of Traditional Chinese Medicine, Shenyang 110847, China

**Keywords:** cycloastragenol, cardiac remodeling, epidermal growth factor receptor, MAPK signaling pathway, autophagy

## Abstract

**Background:** Pathological cardiac remodeling is a major driver of heart failure progression and is closely associated with fibroblast activation, myocardial inflammation, oxidative injury, and impaired autophagic homeostasis. Epidermal growth factor receptor (EGFR) signaling participates in adverse remodeling through activation of MAPK pathways, but the relationship between pharmacologically modulated EGFR turnover and cardiac remodeling remains insufficiently defined. **Methods:** This study investigated the protective effects of cycloastragenol (CAG), a triterpenoid sapogenin derived from Astragalus membranaceus, in Angiotensin II (Ang II)-induced cardiac remodeling and examined changes in EGFR turnover and associated MAPK signaling and autophagy-related processes. Circulating soluble EGFR was associated with heart failure severity after adjustment for age and sex. Separately, an independent exploratory transcriptomic analysis of left ventricular samples from GSE161472 showed that myocardial EGFR expression distinguished HFrEF from non-failing samples with an AUC of 0.870. Male C57BL/6 mice subjected to continuous Ang II infusion at 500 ng/kg/min for 28 days were used to establish the in vivo cardiac remodeling model and evaluate the effects of CAG on cardiac function, myocardial injury, fibrosis, inflammation, oxidative stress, apoptosis, and autophagy-related changes, with enalapril used as a positive control. Primary cardiac fibroblasts were further used to assess proliferation, migration, collagen production, EGFR membrane accumulation, lysosomal trafficking, MAPK signaling, and autophagic flux. Circulating soluble EGFR increased with heart failure severity and was associated with BNP elevation, ventricular dilation, and reduced ejection fraction. **Results:** CAG improved cardiac performance, reduced myocardial injury markers, alleviated fibrosis and hypertrophy, and suppressed inflammatory and oxidative responses in Ang II-treated mice. In cardiac fibroblasts, CAG reduced sustained EGFR membrane accumulation, increased EGFR recovery in pan-ubiquitin immunoprecipitates and lysosomal localization, and accelerated EGFR protein turnover, consistent with a predominantly lysosome-associated pathway. The K716R mutation attenuated CAG-associated changes in EGFR turnover, MAPK phosphorylation, and autophagic flux. **Conclusions:** These findings suggest that CAG attenuates Ang II-induced cardiac remodeling and is associated with enhanced EGFR lysosomal turnover, accompanied by changes in MAPK signaling and autophagy-related processes.

## 1. Introduction

Heart failure (HF) remains a leading cause of morbidity and mortality worldwide, placing a significant clinical and socioeconomic burden on healthcare systems. It is reported that over 64 million individuals are affected by HF globally, and this number continues to rise [1]. At the core of HF pathogenesis lies adverse cardiac remodeling, a maladaptive process marked by cardiomyocyte hypertrophy, interstitial fibrosis, inflammatory infiltration, and progressive left ventricular dilation and dysfunction [2]. Among these pathological features, myocardial fibrosis is particularly detrimental, as it compromises ventricular compliance, impairs systolic and diastolic function, and promotes arrhythmogenesis, ultimately driving the transition to overt heart failure [3]. Although current guideline-directed medical therapies have improved outcomes, they primarily slow disease progression rather than reverse established structural damage [4,5,6]. Furthermore, a substantial subset of patients remains unresponsive or intolerant to these therapies, underscoring the urgent need for novel therapeutic targets and interventions capable of reversing pathological remodeling.

Emerging evidence points to the epidermal growth factor receptor (EGFR) as an important regulator in cardiovascular pathology. EGFR, a transmembrane receptor tyrosine kinase, can be activated or transactivated by mechanical stress and neurohumoral stimuli such as angiotensin II (Ang II) [7,8]. In pathological cardiac remodeling, EGFR signaling is particularly relevant in cardiac fibroblasts, which are major effector cells responsible for fibrotic expansion and adverse structural remodeling [9,10]. Recent studies further demonstrated that EGFR-mediated paracrine signaling promotes stromal activation in hypertrophic cardiomyopathy and that enhanced EGFR phosphorylation contributes to cardiac fibroblast activation and extracellular matrix deposition in diabetic cardiomyopathy [11,12]. Upon activation, EGFR engages multiple downstream signaling pathways, including the mitogen-activated protein kinase (MAPK), PI3K/Akt, TGF-β/Smad, and NF-κB pathways. Among these signaling branches, the MAPK cascade, comprising ERK, JNK, and p38, was selected as the primary focus of the present study because it is closely involved in cardiac fibroblast proliferation, migration, activation, extracellular matrix production, inflammatory responses, and stress-induced cell injury. Through downstream transcription factors, MAPK signaling regulates biological processes that contribute to hypertrophy, fibrosis, inflammation, and cell survival, which are major features of adverse cardiac remodeling [13,14,15]. Beyond its canonical signaling functions, EGFR also interfaces with the autophagy machinery [16,17,18], a cellular degradation system essential for maintaining protein and organelle homeostasis [19,20]. Sustained MAPK signaling has been associated with impaired autophagic homeostasis, intracellular stress, and fibrotic remodeling [21]. Recent studies have further emphasized that autophagy is dynamically regulated during cardiomyopathy progression and that restoration of autophagic homeostasis may facilitate reverse cardiac remodeling. However, the functional consequences of autophagy appear to depend on disease stage, cellular context, and the magnitude and duration of autophagic activity [22,23]. Therefore, investigating MAPK activation provides a mechanistic framework for understanding how changes in EGFR regulation are linked to cardiac fibroblast activation and autophagy-related alterations.

In this context, cycloastragenol (CAG)—a major isoflavonoid glycoside derived from Astragalus membranaceus—has attracted attention due to its potent antioxidant, anti-inflammatory, and anti-fibrotic properties in diverse disease models, including myocardial injury and metabolic disorders [24,25]. Previous studies have shown that CAG can modulate MAPK signaling and enhance autophagic activity, although its direct interaction with EGFR has not been fully elucidated [26].

Based on these findings, we investigated whether the cardioprotective effects of CAG are associated with alterations in EGFR turnover, MAPK signaling, and autophagy-related processes during pathological cardiac remodeling. To test this, we employed a murine model of Ang II-induced cardiac remodeling, in which enalapril, a clinically established angiotensin-converting enzyme inhibitor widely used in heart failure management and known to attenuate cardiac fibrosis and adverse remodeling [27], was included as a pharmacological positive control to provide a clinically relevant benchmark. In parallel, multiple doses of CAG were applied to evaluate its dose-dependent efficacy and determine whether its protective effects could reach or exceed those of a standard therapeutic agent. Our study provides new insights into the relationship between the cardioprotective effects of CAG and changes in EGFR turnover, MAPK signaling, and autophagy-related processes during pathological cardiac remodeling.

## 2. Results

### 2.1. EGFR Upregulation May Be Involved in the Progression of Adverse Cardiac Remodeling

**Figure 1:**
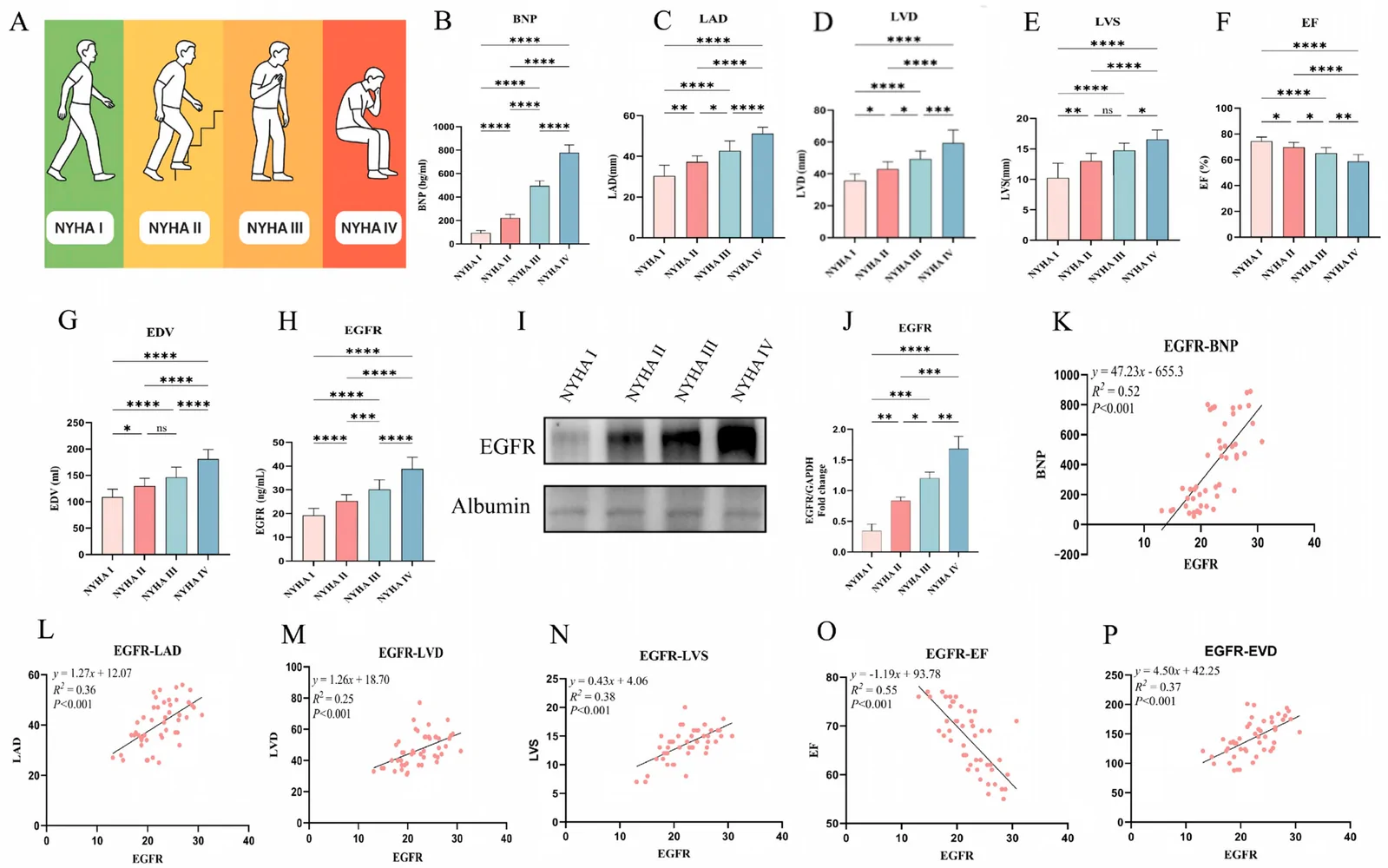
Elevated EGFR expression correlates with NYHA class progression and cardiac dysfunction. (**A**) Schematic illustration of NYHA functional classification stages I–IV. (**B**–**G**) Plasma levels of BNP and echocardiographic parameters (LAD, LVD, LVS, EF, and EDV) across NYHA I–IV groups (*n* = 12 per group). (**H**) Representative Western blot showing EGFR expression in peripheral blood samples. (**I**) Quantification of EGFR protein levels revealed a stepwise increase corresponding to advancing NYHA class. (**J**–**O**) Pearson correlation analysis showing that plasma EGFR levels positively correlated with BNP (**K**), LAD (**L**), LVD (**M**), LVS (**N**), and EDV (**P**) and negatively correlated with EF (**O**). Data are presented as mean ± SD. ns, not significant; *p*  <  0.05 (*), *p*  <  0.01 (**), *p*  <  0.001 (***), *p*  <  0.0001 (****).

To investigate the potential involvement of circulating EGFR in adverse cardiac remodeling, we enrolled 48 patients with chronic heart failure and stratified them into four groups according to the New York Heart Association (NYHA) functional classification (*n* = 12 per group) (Figure 1A). Baseline clinical characteristics, including age, sex, smoking, alcohol consumption, comorbidities, and medication use, showed no statistically significant differences among the groups (all *p* > 0.05; Table 1).

Echocardiographic and biochemical analyses showed progressive deterioration in cardiac function across the NYHA classes. BNP, LAD, LVD, LVS, and EDV increased from NYHA class I to IV, whereas EF progressively decreased (Figure 1B–G), consistent with increasing ventricular dilation and systolic dysfunction.

To assess the association between circulating EGFR and heart failure severity, circulating soluble EGFR levels were measured using ELISA and Western blotting. Serum EGFR concentrations increased progressively across the NYHA classes, consistent with the Western blot results showing higher EGFR protein abundance in patients with more advanced heart failure (Figure 1H–J). Correlation analysis showed that circulating EGFR levels were positively correlated with BNP (r = 0.52), LAD (r = 0.36), LVD (r = 0.25), LVS (r = 0.38), and EDV (r = 0.37) and negatively correlated with EF (r = −0.55) (Figure 1K–P).

Multivariable regression analyses further showed that circulating EGFR remained significantly associated with LVEF, ln(BNP), LVD, LAD, IVS, and EDV after adjustment for age and sex (all *p* < 0.001; Figure S4F). Separately, as an independent exploratory transcriptomic analysis, ROC analysis of myocardial EGFR expression in left ventricular samples from GSE161472 showed discrimination between HFrEF and non-failing samples, with an AUC of 0.870 (Figure S4G). Together, the clinical cohort data support an association between circulating soluble EGFR and heart failure severity, whereas the GSE161472 analysis independently provides exploratory transcriptomic evidence for EGFR-related alterations in failing myocardium.

### 2.2. CAG Mitigates AngII-Induced Cardiac Remodeling, Hypertrophy, Fibrosis, and Myocardial Injury

**Figure 2:**
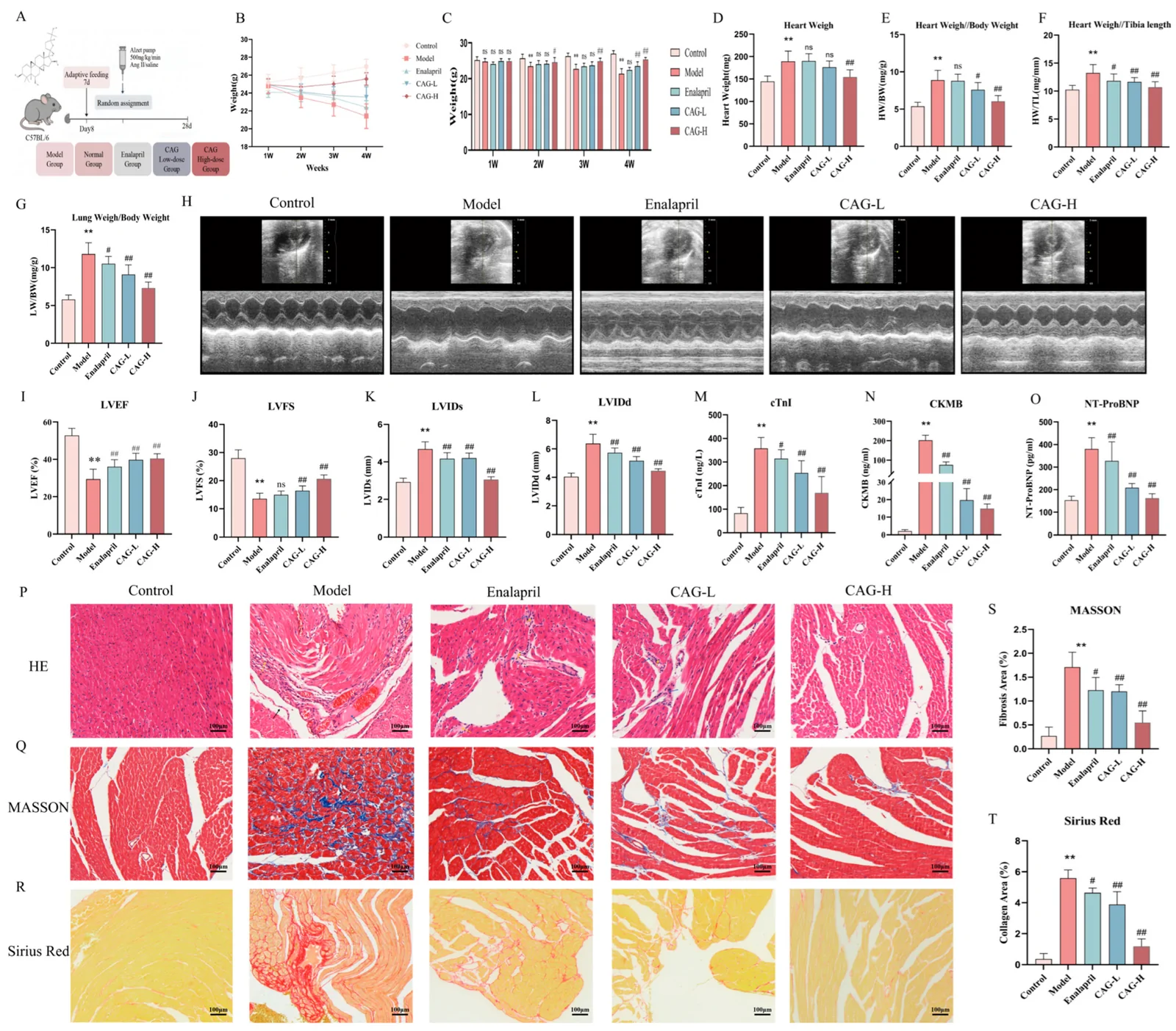
CAG attenuates Ang II-induced cardiac hypertrophy and fibrosis. (**A**) Experimental protocol: mice were infused with Ang II for 28 days and treated with low- or high-dose CAG. (**B**,**C**) Body weight trends show that CAG, particularly at high dose, alleviated Ang II-induced weight loss. (**D**–**G**) HW/BW, HW/TL, and HW/LW were significantly increased in the model group and dose-dependently reduced by CAG treatment. (**H**–**L**) Representative M-mode echocardiographic images and quantitative assessment of LVEF, LVFS, LVIDs, and LVIDd in control, model, and CAG-treated groups. (**M**–**O**) Serum levels of NT-proBNP, cTnI, and CKMB detected by ELISA to evaluate myocardial injury. (**P**) HE staining shows normal myocardial architecture in the control group, while the model group displays myocyte necrosis (blue arrows), inflammatory infiltration (yellow arrows), and fiber disarray. CAG treatment restored histological structure in a dose-dependent manner. (**Q**,**S**) Masson’s trichrome and Sirius Red staining reveal marked collagen deposition in the model group, which was significantly reduced by CAG treatment. (**R**,**T**) Quantification of fibrotic area and collagen-positive area further confirmed the anti-fibrotic effects of CAG. Data are presented as mean ± SD (*n* = 5). ns, not significant; ** *p* < 0.01 vs. control group; # *p* < 0.05, ## *p* < 0.01 vs. model group.

To evaluate the cardioprotective effects of CAG against AngII-induced pathological cardiac remodeling and functional impairment, we established a mouse model with continuous AngII infusion for 28 days (Figure 2A). The model group exhibited significant weight loss, cardiac hypertrophy, and impaired cardiac function, as evidenced by increased heart weight-to-body weight ratio (HW/BW), heart weight-to-lung weight ratio (HW/LW), reduced LVEF and LVFS, and increased LVIDd and LVIDs. To assess myocardial injury, serum levels of cTnI, CKMB, and NT-ProBNP were measured. All three biomarkers were markedly elevated in the model group, reflecting substantial myocardial damage. In contrast, CAG treatment significantly attenuated weight loss, reduced cardiac hypertrophy, and improved cardiac function, with the most pronounced effects in the CAG-H group (Figure 2B–L). Moreover, CAG treatment significantly reduced the levels of cTnI, CKMB, and NT-ProBNP, with the largest decreases observed in the CAG-H group (Figure 2M–O). Enalapril also demonstrated improvement but to a lesser extent. Histological analyses showed that CAG treatment markedly reduced myocardial fibrosis, as seen by Masson’s trichrome and Sirius Red staining, and alleviated pathological myocardial changes, including disorganized myocardial fibers, inflammatory cell infiltration, and focal necrosis (Figure 2P–T).

Additionally, to assess oxidative stress and apoptosis, we performed ROS and TUNEL staining. The model group exhibited markedly elevated myocardial ROS levels at the tissue level, significant cardiomyocyte apoptosis, and abnormal cardiomyocyte morphology, indicating enhanced oxidative stress, increased cell death, and cardiomyocyte hypertrophy. CAG treatment significantly reduced ROS levels, suppressed cardiomyocyte apoptosis, and improved cardiomyocyte morphology, with the most pronounced improvements observed in the CAG-H group (Figure S1A–F).

In summary, CAG—particularly at high doses—effectively mitigated oxidative stress, suppressed cardiomyocyte apoptosis, attenuated myocardial fibrosis, and improved cardiac function in AngII-induced cardiac remodeling. CAG demonstrated superior cardioprotective efficacy compared to enalapril, with the most pronounced benefits observed in the CAG-H group.

### 2.3. CAG Suppresses Cardiac Inflammation and Fibrotic Remodeling in AngII-Induced Heart Injury

Based on metabolomic analysis, we hypothesized that CAG may reverse disease-associated metabolic reprogramming by modulating autophagy, the ErbB signaling pathway, and the MAPK signaling pathway. These pathways are known to be closely linked to myocardial inflammation, fibrosis, and structural remodeling. To validate this, we conducted histological and molecular experiments to assess the effects of CAG on inflammation and fibrosis, two core pathological processes driving cardiac remodeling.

**Figure 3:**
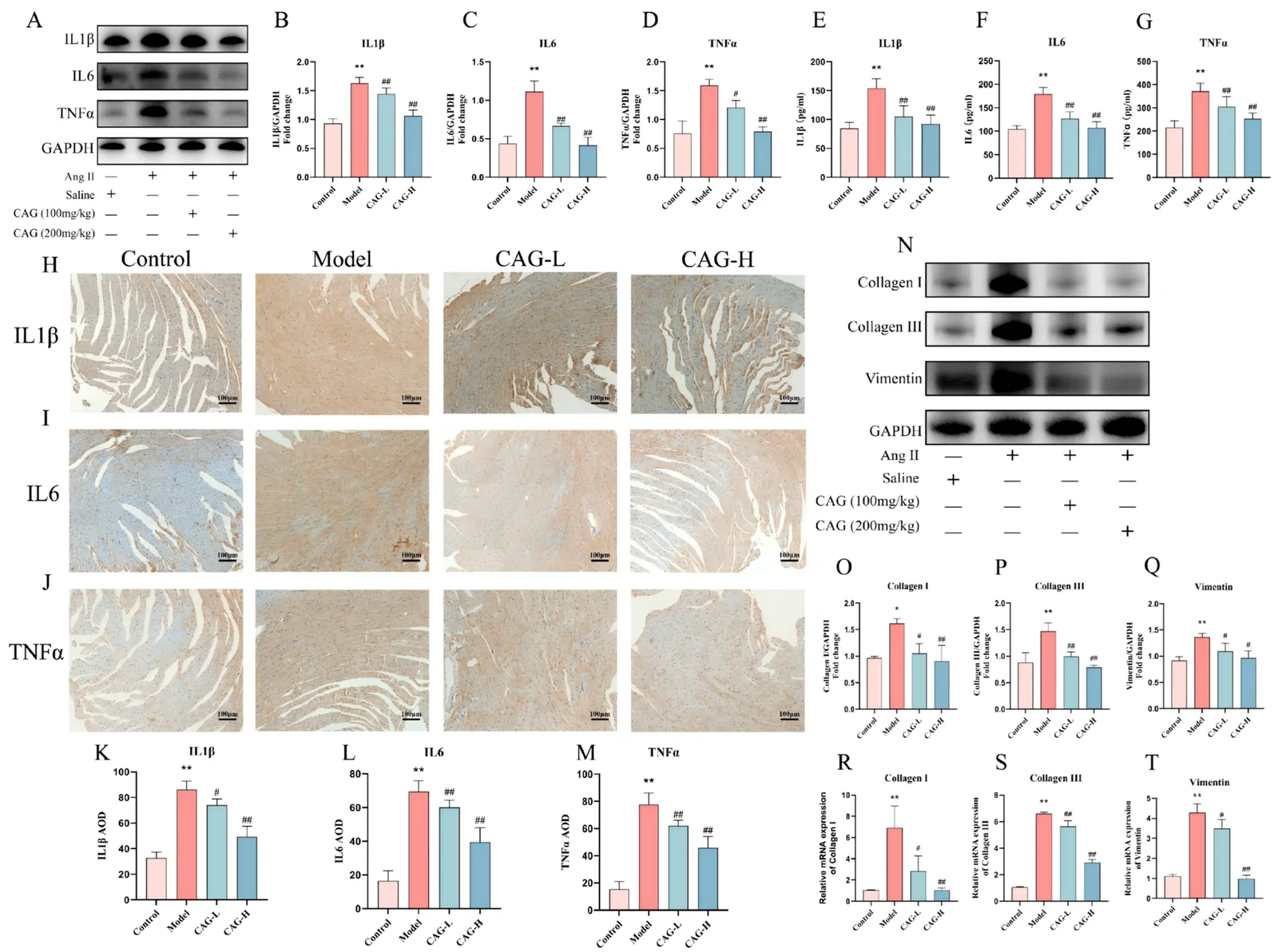
CAG alleviates cardiac inflammation and fibrosis in AngII-induced cardiac remodeling. (**A**–**D**) Western blot showing IL-1β, IL-6, and TNF-α expression. (**E**–**G**) ELISA showing IL-1β, IL-6, and TNF-α expression. (**H**–**M**) IHC staining and quantification of IL-1β, IL-6, and TNF-α. (**N**–**T**) Western blot and qPCR of Collagen I, Collagen III, and Vimentin. Data are presented as mean ± SD (*n* = 5/3). * *p* < 0.05, ** *p* < 0.01 vs. control; # *p* < 0.05, ## *p* < 0.01 vs. model.

Western blot and ELISA analyses revealed that CAG markedly suppressed the AngII-induced upregulation of IL-1β, IL-6, and TNF-α protein expression in myocardial tissue (Figure 3A–G). IHC analysis further confirmed that CAG treatment significantly attenuated myocardial inflammatory signals, as evidenced by reduced staining intensity and lower average optical density (Figure 3H–M). Regarding fibrosis, CAG treatment substantially reduced AngII-induced expression of Collagen I, Collagen III, and Vimentin proteins (Figure 3N–Q), with corresponding mRNA expression showing a similar pattern (Figure 3R–T).

Collectively, these results demonstrate that CAG effectively alleviates myocardial inflammation and inhibits fibrotic remodeling, thereby contributing to its overall cardioprotective effects.

### 2.4. CAG Reduces EGFR Abundance and Modulates MAPK Signaling and Autophagy-Related Changes

**Figure 4:**
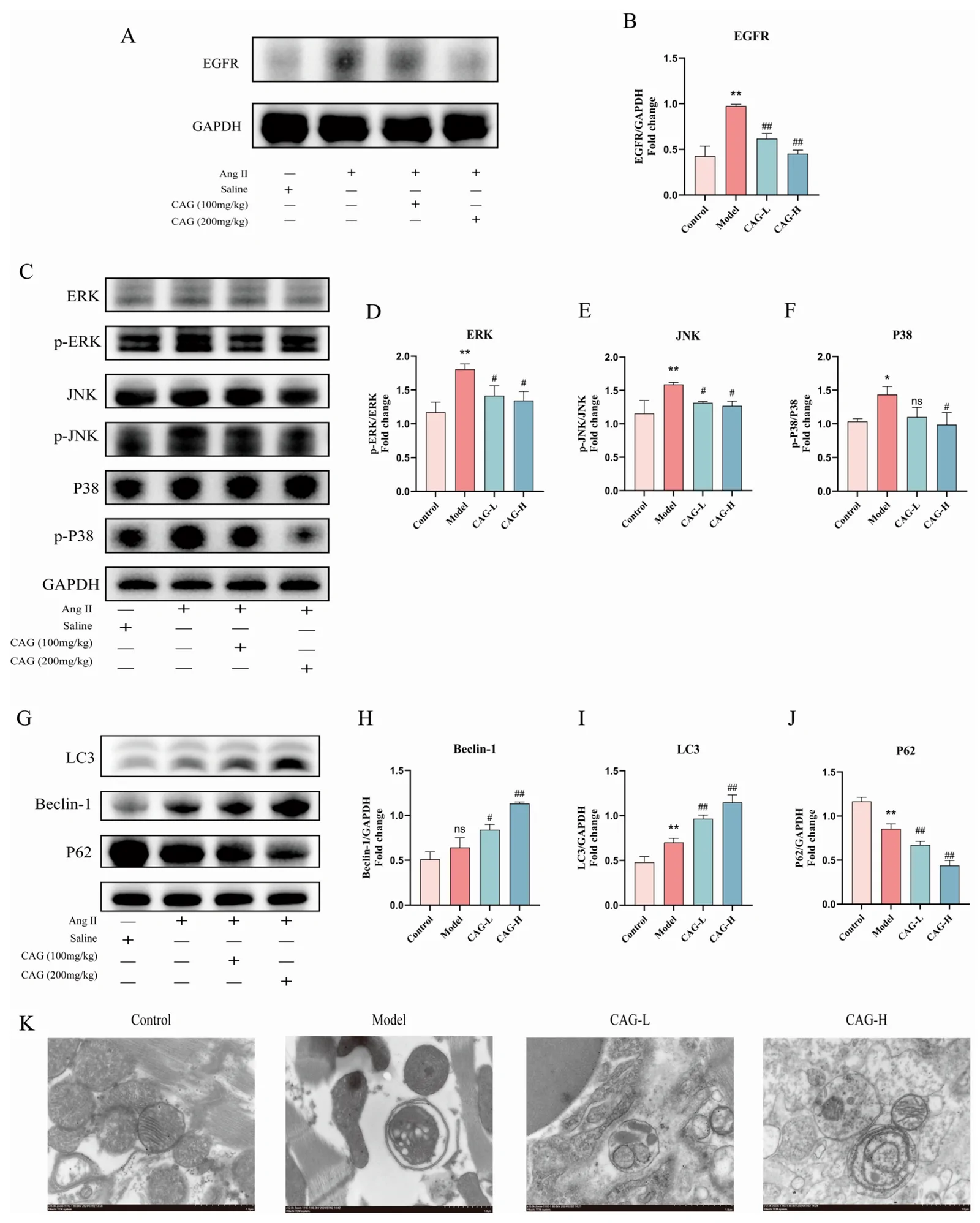
CAG treatment is associated with reduced EGFR abundance, lower MAPK phosphorylation, and autophagy-related changes in cardiac tissue. (**A**) Western blot of EGFR. (**B**) Quantitative analysis of EGFR Western blotting. (**C**) Western blot of phosphorylated and total ERK, JNK, and P38. (**D**–**F**) Ratio analysis of phosphorylated/total ERK, JNK, and P38. (**G**) Western blot analysis of LC3, Beclin-1, and P62 expression. (**H**–**J**) Quantification of LC3, Beclin-1, and P62 protein levels. (**K**) TEM images showing autophagic vesicles. Data are shown as mean ± SD (*n* = 5/3). ns, not significant; * *p* < 0.05, ** *p* < 0.01 vs. control; # *p* < 0.05, ## *p* < 0.01 vs. model.

Based on these findings, we further examined EGFR abundance and associated changes in MAPK signaling and autophagy-related processes during CAG treatment. Western blotting showed that CAG reduced EGFR protein abundance in a dose-dependent manner (Figure 4A,B), while immunofluorescence analysis demonstrated decreased EGFR signals in myocardial tissue (Figure S2A). ELISA further showed that CAG reduced circulating EGFR levels in Ang II-induced heart failure mice (Figure S3E).

We next assessed MAPK phosphorylation at the terminal experimental endpoint. At the end of the Ang II infusion and CAG treatment period, the phosphorylation levels of ERK, JNK, and p38 were significantly increased in the model group and markedly reduced following CAG treatment (Figure 4C–F). These findings indicate that the CAG-associated reduction in EGFR abundance was accompanied by lower ERK, JNK, and p38 phosphorylation at the terminal assessment.

Because EGFR-associated signaling is also involved in autophagy regulation, we further evaluated autophagy-related changes. CAG treatment increased LC3 and Beclin-1 expression and reduced p62 levels (Figure 4G–J). Immunofluorescence and transmission electron microscopy further showed increased autophagy-associated signals and restoration of characteristic autophagosomal structures (Figure 4K and Figure S2B). Together, these static molecular and morphological findings support the modulation of autophagy-related processes by CAG in vivo but do not independently establish restoration of autophagic flux.

### 2.5. CAG Suppresses Ang II-Induced Fibroblast Activation, Proliferation, Migration, and Fibrosis In Vitro

To investigate whether CAG directly affects cardiac fibroblasts in myocardial fibrosis, we conducted in vitro experiments. In our previous in vivo studies, we demonstrated that CAG attenuates myocardial inflammation and fibrosis and modulates EGFR, MAPK signaling, and autophagic activity. However, due to the complexity of the in vivo environment, it remains difficult to determine its direct effects on the key effector cells driving cardiac remodeling. Cardiac fibroblasts, which are responsible for myocardial fibrosis, play a key role in this process through their proliferation, migration, and activation.

**Figure 5:**
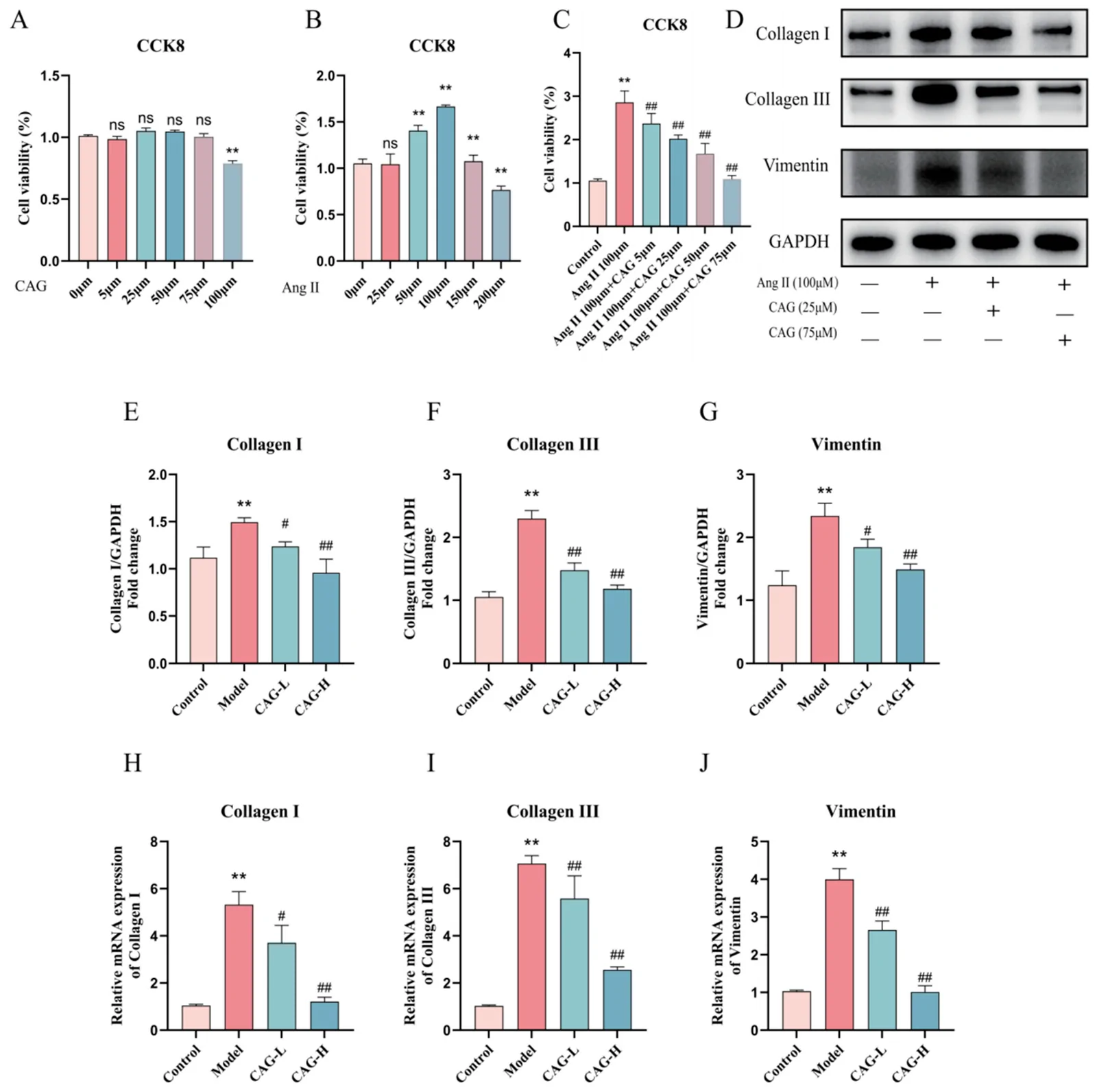
CAG suppresses Ang II-induced fibroblast proliferation, migration, colony formation, fibrotic marker expression, and cytoskeletal remodeling. (**A**–**C**) CCK-8 assay showing cell viability after Ang II stimulation, CAG treatment alone, and Ang II + CAG co-treatment. (**D**) Western blot analysis of Collagen I, Collagen III, and Vimentin protein levels in each group. (**E**–**G**) Quantification of Collagen I, Collagen III, and Vimentin protein expression. (**H**–**J**) Relative mRNA expression of Collagen I, Collagen III, and Vimentin assessed by RT-qPCR. Data are presented as mean ± SD (*n* = 5/3). ns, not significant; ** *p* < 0.01 vs. control; # *p* < 0.05, ## *p* < 0.01 vs. model.

We treated fibroblasts with varying doses of Ang II and CAG to identify optimal concentrations for in vitro modeling. CCK8 assays revealed that Ang II promoted fibroblast viability in a dose-dependent manner, with 100 μm having the most pronounced effect (Figure 5B). When administered alone, CAG showed no cytotoxicity up to 75 μm, while 100 μm significantly reduced cell viability, suggesting potential toxicity (Figure 5A). We found that CAG suppressed Ang II-induced fibroblast activation in a dose-dependent manner. At 25 μm (CAG-L), CAG significantly inhibited fibroblast activation, and at 75 μm (CAG-H), the effect was even more pronounced, reducing fibroblast viability to near-control levels (Figure 5C). Further functional assays supported these findings. CAG treatment significantly reduced fibroblast proliferation, migration, and clonogenicity. EdU staining showed a decrease in the proportion of proliferating cells (Figure S2C), wound-healing assays showed inhibited migration (Figure S2D), and colony formation assays revealed a marked reduction in colony number and size (Figure S2E).

To assess the impact of CAG on fibrosis at the molecular level, we conducted a fibrosis assay. Western blot analysis revealed that Ang II markedly increased the protein levels of Collagen I, Collagen III, and Vimentin, while CAG attenuated these increases in a dose-dependent manner (Figure 5D–G). RT-qPCR showed that CAG also reduced the mRNA expression of these fibrosis-related genes (Figure 5H–J). Since cytoskeletal remodeling is crucial in fibroblast activation and fibrotic progression, we assessed F-actin organization by phalloidin staining. Ang II treatment induced pronounced F-actin accumulation and the formation of thick stress fibers, whereas CAG significantly reduced fluorescence intensity and diminished F-actin aggregation (Figure S2F).

Collectively, these findings demonstrate that CAG effectively suppresses Ang II-induced fibroblast proliferation, migration, clonogenicity, fibrosis, and cytoskeletal remodeling, supporting its potential role in inhibiting myocardial fibrosis through direct effects on cardiac fibroblasts.

### 2.6. CAG Treatment Is Associated with Changes in Ubiquitin-Associated EGFR Signaling, MAPK Signaling, and Autophagic Flux in Ang II-Stimulated Cardiac Fibroblasts

Ubiquitination is a key post-translational modification that regulates EGFR stability and signaling by directing it to degradation pathways. EGFR ubiquitination is involved in receptor stability and intracellular trafficking and may influence downstream signaling processes. Therefore, we examined the ubiquitin-associated EGFR signal together with MAPK signaling and autophagic flux following CAG treatment.

**Figure 6:**
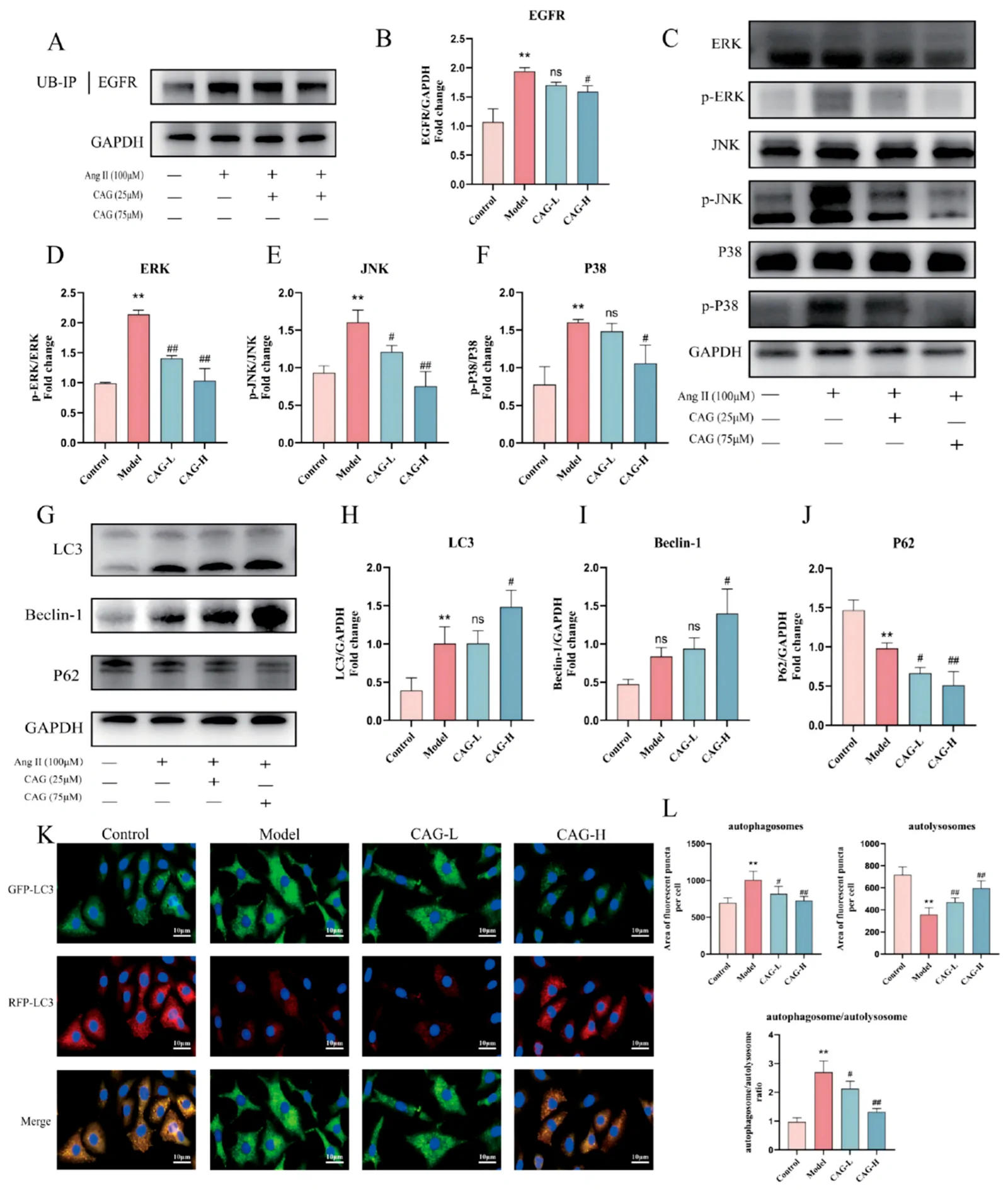
CAG treatment is associated with increased ubiquitin-associated EGFR signal, reduced MAPK phosphorylation, improved autophagic flux, and attenuated fibrotic phenotypes in Ang II-stimulated fibroblasts. (**A**) Pan-ubiquitin immunoprecipitation followed by EGFR immunoblotting showing EGFR recovery in each group. (**B**) Quantification of EGFR signal recovered in pan-ubiquitin immunoprecipitates. (**C**) Western blot analysis of phosphorylated and total ERK, JNK, and P38. (**D**–**F**) Densitometric quantification of phosphorylated/total ratios for ERK, JNK, and P38. (**G**) Western blot analysis of autophagy-related proteins LC3, Beclin-1, and P62 in different treatment groups. (**H**–**J**) Quantification of LC3, Beclin-1, and P62 ratios, respectively. (**K**) Representative images of tandem fluorescent-tagged LC3 (mRFP-GFP-LC3) to monitor autophagic flux. GFP (green) indicates autophagosomes; RFP (red) indicates both autophagosomes and autolysosomes. DAPI staining (blue) indicates cell nuclei. (**L**) Quantification of fluorescent puncta per cell for GFP (autophagosomes), RFP (autolysosomes), and autophagosome/autolysosome ratio. Data are presented as mean ± SEM (*n* = 5/3). ns, not significant; ** *p* < 0.01 vs. control; # *p* < 0.05, ## *p* < 0.01 vs. model.

We therefore examined changes in the ubiquitin-associated EGFR signal, MAPK signaling, and autophagic flux in Ang II-stimulated cardiac fibroblasts following CAG treatment. Pan-ubiquitin immunoprecipitation followed by EGFR immunoblotting showed treatment-related differences in EGFR recovery in the immunoprecipitated fraction. CAG, particularly at high doses, increased the EGFR signal detected in pan-ubiquitin immunoprecipitates (Figure 6A,B), with immunofluorescence confirming reduced EGFR fluorescence intensity after CAG treatment (Figure S2G). At the 24 h treatment endpoint, Ang II increased the phosphorylation ratios of ERK, JNK, and p38, whereas CAG reduced these phosphorylation levels in a dose-dependent manner (Figure 6C–F).

Autophagy evaluation showed that CAG upregulated LC3 and Beclin-1 while downregulating P62, indicating enhanced autophagy initiation (Figure 6G–J). MDC staining confirmed increased autophagosomes with high-dose CAG (Figure S2H), and the mRFP-GFP-LC3 assay showed that CAG restored autophagic flux, reducing GFP puncta and increasing RFP puncta (Figure 6K,L).

In summary, CAG treatment was associated with reduced fibroblast activation, increased ubiquitin-associated EGFR signal, lower MAPK phosphorylation at the 24 h endpoint, and improved autophagic flux.

### 2.7. K716R Attenuates CAG-Associated Changes in EGFR Turnover, MAPK Signaling, and Autophagy

**Figure 7:**
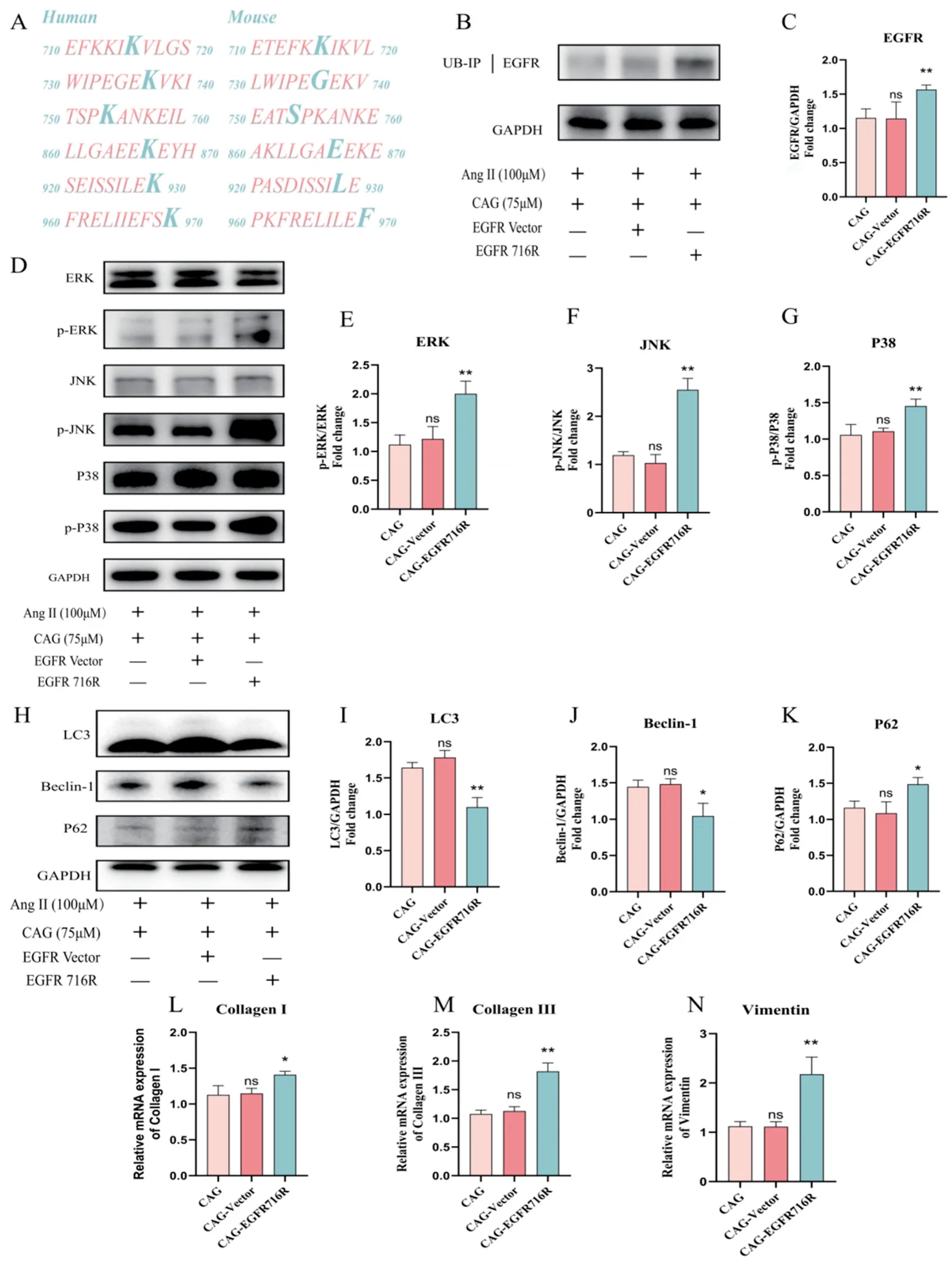
Exploratory analysis of the effects of EGFR K716R substitution on CAG-associated cellular responses. (**A**) Amino acid sequence alignment of EGFR from human and mouse tissue, highlighting the conserved lysine 716 site. (**B**,**C**) Pan-ubiquitin immunoprecipitation followed by EGFR immunoblotting and corresponding quantification, showing that the CAG-associated increase in ubiquitin-associated EGFR signal was attenuated by K716R. (**D**–**G**) Western blot analysis and quantification of phosphorylated/total ERK, JNK, and P38 MAPK proteins. (**H**–**K**) Western blot and quantification of autophagy-related proteins LC3, Beclin-1, and P62. (**L**–**N**) Relative mRNA expression levels of Collagen I, Collagen III, and Vimentin in the three groups. Data are shown as mean ± SD (*n* = 5/3). * *p* < 0.05, ** *p* < 0.01 vs. CAG or CAG-Vector; ns, not significant.

Previous proteomic studies have identified multiple ubiquitin-acceptor lysine residues within the intracellular region of EGFR, including K716 [28,29], and previous functional studies have reported that K716R substitution can alter overall EGFR ubiquitination and protein stability [30,31]. Based on these previous observations and the conservation of K716 between human and mouse EGFR, K716 was selected as one literature-guided candidate residue for exploratory mutational analysis. This selection was not intended to imply that K716 is the predominant, unique, or direct ubiquitin conjugation site of EGFR. Sequence alignment confirmed conservation of this residue between human and mouse EGFR (Figure 7A). We therefore used the K716R substitution to examine whether alteration of this candidate residue was associated with changes in CAG-related EGFR regulation. Accordingly, K716 was examined as one literature-guided exploratory candidate residue, rather than as an established principal or unique ubiquitination site. We generated the K716R mutant to assess whether alteration of this residue modifies CAG-associated EGFR regulation, MAPK signaling, and autophagy-related changes.

Pan-ubiquitin immunoprecipitation followed by EGFR immunoblotting showed that CAG increased the ubiquitin-associated EGFR signal in cells expressing EGFR-WT, whereas this increase was attenuated in cells expressing EGFR-K716R (Figure 7B,C). At the 24 h treatment endpoint, the K716R mutation attenuated the CAG-associated reductions in ERK, JNK, and p38 phosphorylation (Figure 7D–G). Furthermore, the K716R mutation suppressed autophagy, as indicated by reduced LC3 and Beclin-1 expression and increased P62 levels (Figure 7H–K). mRFP-GFP-LC3 assays confirmed that the K716R mutation impaired autophagic flux, increasing autophagosome formation and reducing autolysosome formation (Figure S2I).

To further examine whether alteration of K716 was associated with the cellular responses observed following CAG treatment, fibroblasts expressing EGFR-WT or EGFR-K716R were evaluated for proliferation, migration, cytoskeletal organization, and fibrosis-related markers. The K716R mutation significantly restored fibroblast migration, proliferation, and colony formation, which were inhibited by CAG (Figure S3A,B,D). Additionally, cytoskeletal analysis revealed increased F-actin polymerization in the K716R group (Figure S3C), and Collagen I, Collagen III, and Vimentin were upregulated, reversing CAG’s effects (Figure 7L–N). In summary, the K716R substitution attenuated several CAG-associated changes in EGFR regulation, MAPK phosphorylation, autophagic flux, and fibroblast phenotypes. These findings indicate that alteration of K716 can modify several CAG-associated responses; however, they do not establish K716 as a direct, predominant, or unique ubiquitin conjugation site, nor do they demonstrate that K716 is required for the cardioprotective effects of CAG.

### 2.8. CAG Enhances EGFR Lysosomal Turnover and Is Accompanied by Reduced MAPK-Responsive Transcriptional Output

In our previous experiments, CAG increased the EGFR signal recovered in pan-ubiquitin immunoprecipitates and reduced EGFR protein abundance, whereas K716R attenuated these changes. However, whether CAG accelerated EGFR protein turnover and which degradation pathway predominantly contributed to this process remained to be determined.

**Figure 8:**
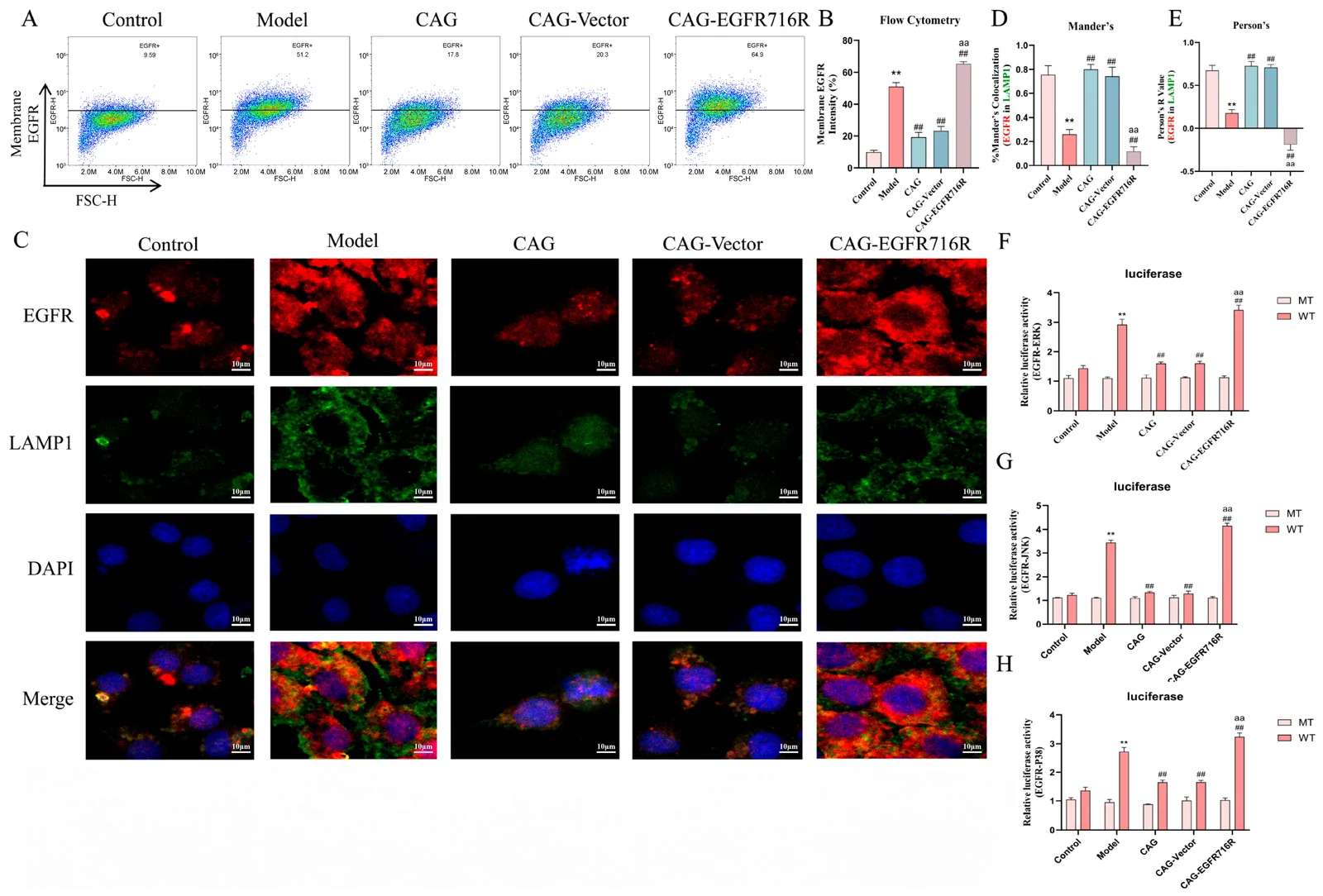
CAG-associated EGFR lysosomal turnover and concomitant changes in MAPK-responsive reporter activities. (**A**,**B**) Flow cytometry showing that CAG reduced surface EGFR expression, while the 716R mutation reversed this effect. (**C**) Immunofluorescence images of EGFR (red), LAMP1 (green), and nuclei (DAPI, blue) showing subcellular localization. (**D**,**E**) Quantification of colocalization using Pearson and Manders’ coefficients. (**F**–**H**) Dual-luciferase reporter assays showing that CAG reduced ERK/Elk-1-, JNK/c-Jun-, and p38/CHOP-responsive reporter activities, whereas these effects were attenuated by the EGFR-K716R mutation. Data are shown as mean ± SD (*n* = 5/3). ** *p* < 0.01 vs. Control group; ## *p* < 0.01 vs. Model group; aa < 0.01 vs. CAG group.

To address this issue, we first quantified cell-surface EGFR by flow cytometry. Ang II stimulation increased membrane-associated EGFR abundance in cardiac fibroblasts, whereas CAG treatment significantly reduced cell-surface EGFR levels. This effect was attenuated by the K716R mutation (Figure 8A,B), suggesting that K716 is functionally involved in the regulation of EGFR membrane accumulation and intracellular trafficking.

We subsequently examined the colocalization of EGFR with the lysosomal marker LAMP1. CAG increased EGFR–LAMP1 colocalization, consistent with enhanced trafficking of EGFR toward lysosomal compartments, whereas the K716R mutation attenuated this effect (Figure 8C–E). ELISA further showed that Ang II increased soluble EGFR accumulation in the culture supernatant, whereas CAG reduced soluble EGFR levels. This effect was also attenuated by the K716R mutation (Figure S3F). Because the source and release mechanism of extracellular soluble EGFR were not directly examined, these findings were interpreted as changes in soluble EGFR accumulation rather than direct evidence of altered EGFR secretion.

To determine the degradation pathway contributing to the CAG-induced reduction in EGFR, cardiac fibroblasts were treated with the proteasome inhibitor MG132 or the lysosomal inhibitor BafA1. CAG significantly reduced EGFR protein abundance in Ang II-stimulated cells. BafA1 markedly reversed this reduction, whereas MG132 produced no significant restorative effect (Figure S4A,B). These findings support a predominantly lysosome-associated mechanism of EGFR degradation following CAG treatment.

We further performed cycloheximide-chase assays to evaluate EGFR protein stability and turnover. Following inhibition of de novo protein synthesis, EGFR remained relatively stable in Ang II-stimulated cells during the 24 h observation period, whereas CAG accelerated its time-dependent reduction. Expression of EGFR-WT retained the CAG-associated turnover response, whereas the K716R mutation delayed EGFR reduction (Figure S4C,D). Nonlinear regression analysis further showed that CAG shortened the apparent EGFR protein half-life, whereas K716R increased EGFR stability relative to EGFR-WT (Figure S4E).

Collectively, the flow-cytometric, EGFR–LAMP1 colocalization, pharmacological inhibition, CHX-chase, ELISA, and pan-ubiquitin IP results indicate that CAG reduces cell-surface EGFR accumulation and is associated with enhanced EGFR lysosomal trafficking and turnover, together with an increased ubiquitin-associated EGFR signal. The attenuation of several of these changes by K716R indicates that alteration of this residue can influence the CAG-associated regulatory responses.

We next assessed the downstream transcriptional responses associated with MAPK signaling. Dual-luciferase reporter assays showed that Ang II increased ERK/Elk-1-, JNK/c-Jun-, and p38/CHOP-responsive reporter activities, whereas CAG significantly reduced these downstream reporter signals. The inhibitory effects of CAG were attenuated by the K716R mutation (Figure 8F–H). These findings indicate that the CAG-associated changes in EGFR turnover and the functional effects of the K716R mutation were accompanied by changes in MAPK-responsive transcriptional output.

## 3. Discussion

Cardiac remodeling is a pathological process characterized by structural and functional alterations of the myocardium in response to sustained stress such as hypertension or neurohormonal stimulation [32,33]. It is a major contributor to the progression of heart failure and is associated with increased morbidity and mortality worldwide. Despite advances in pharmacological management, current therapeutic strategies—mainly targeting neurohormonal pathways—often fail to fully halt or reverse remodeling, highlighting the urgent need for novel molecular targets and regulatory mechanisms. Previous studies have identified EGFR as a key modulator of pathological cardiac remodeling [34]; however, its clinical relevance and underlying mechanisms remain incompletely understood. Using clinical echocardiographic evaluation combined with biochemical profiling, we found that circulating soluble EGFR levels were significantly elevated in heart failure patients and correlated positively with key indicators of cardiac structural and functional impairment, such as LAD, LVD, EDV, and BNP, and negatively with EF. The persistence of the associations between circulating EGFR and cardiac functional and remodeling indices after adjustment for age and sex further supports its relationship with heart failure severity. Separately, the exploratory analysis of GSE161472 provides independent transcriptomic support for EGFR-related alterations in failing myocardium. Importantly, circulating soluble EGFR and myocardial EGFR transcript levels represent distinct biological layers. Mechanistically, EGFR functions as a proximal activator of the MAPK cascade, a well-characterized signaling pathway implicated in cardiac hypertrophy, fibrosis, and inflammation [35,36,37]. MAPK signaling does not operate independently but interacts with other remodeling-related pathways. PI3K/Akt and ERK signaling can be activated in parallel and cooperatively regulate cardiac fibroblast proliferation and cell-cycle progression [38]. In cardiac fibroblasts, TGF-β1 activates canonical Smad signaling together with PI3K/Akt, ERK1/2, JNK, and p38 MAPK pathways, and these signaling branches jointly participate in extracellular matrix synthesis and collagen maturation [39,40]. Under Ang II stimulation, ROS-dependent p38 MAPK signaling can promote NF-κB activation, whereas ERK1/2 contributes to collagen expression, thereby linking oxidative stress, inflammatory transcription, and pro-fibrotic responses [41]. Thus, MAPK represents an important signaling convergence point linking EGFR activation with fibroblast proliferation, inflammation, and extracellular matrix remodeling. Our in vivo and in vitro experiments showed that Ang II treatment was accompanied by increased EGFR abundance and increased phosphorylation of ERK, JNK, and p38. In parallel, Ang II-induced remodeling was associated with autophagy-related abnormalities, including altered LC3 processing, p62 accumulation, and impaired autophagic flux in cardiac fibroblasts. Autophagy is a critical homeostatic process that preserves cardiac function under stress conditions by facilitating the clearance of damaged proteins, dysfunctional organelles, and excessive cellular components [42]. When autophagy is suppressed, this leads to the accumulation of toxic cellular debris, mitochondrial dysfunction, and elevated oxidative stress, which together contribute to cellular apoptosis and maladaptive cardiac remodeling [43,44]. Thus, impaired autophagy is not only an accompanying alteration in pathological remodeling but also a key driving factor. These observations are consistent with the coexistence of altered EGFR signaling and autophagic dysfunction during pathological cardiac remodeling.

CAG is a triterpenoid sapogenin derived from Astragalus membranaceus and has been reported to exert anti-inflammatory, antioxidant, and anti-fibrotic effects. Previous cardiovascular studies have mainly attributed the protective effects of CAG to the regulation of inflammation, oxidative stress, autophagy, NLRP3 inflammasome activation, and MAPK-related signaling. However, to our knowledge, no previous study has systematically examined whether CAG treatment is associated with altered EGFR intracellular trafficking and lysosomal turnover together with changes in ubiquitin-associated EGFR signaling during pathological cardiac remodeling, which represents a principal novel observation of the present study. In the present study, CAG attenuated Ang II-induced cardiac dysfunction, myocardial fibrosis, inflammation, oxidative stress, and apoptosis. In cardiac fibroblasts, CAG inhibited proliferation, migration, and extracellular matrix production, accompanied by reduced EGFR abundance, suppressed MAPK activation, and improved autophagic flux. CAG increased EGFR recovery in pan-ubiquitin immunoprecipitates, enhanced EGFR lysosomal localization, reduced membrane-associated and extracellular EGFR accumulation, and accelerated EGFR protein turnover. Pharmacological inhibition experiments showed that BafA1, but not MG132, markedly attenuated the CAG-induced reduction in EGFR, supporting a predominantly lysosome-associated degradation mechanism. Mechanistically, ubiquitination-dependent endolysosomal sorting directs activated EGFR toward lysosomal degradation and limits receptor signaling [45]; active EGFR can also bind and tyrosine-phosphorylate Beclin-1, promoting its association with the inhibitory proteins Bcl-2 and Rubicon while reducing Beclin-1-associated VPS34 kinase activity, thereby suppressing autophagy initiation [46]. Accordingly, CAG-associated acceleration of EGFR lysosomal turnover may alleviate this inhibitory input and facilitate autophagic flux, consistent with the combined changes in LC3 processing, p62/SQSTM1 accumulation, and tandem fluorescent LC3 reporter signals observed in this study [47]. In parallel, CAG-associated regulation of EGFR turnover was accompanied by reduced phosphorylation of ERK, JNK, and p38; decreased ERK/Elk-1-, JNK/c-Jun-, and p38/CHOP-responsive reporter activities; and attenuation of fibroblast proliferation, migration, and extracellular matrix production. These findings indicate that CAG-associated regulation of EGFR turnover was accompanied by attenuation of MAPK phosphorylation and downstream MAPK-responsive transcriptional output. K716 was selected as a conserved candidate residue for exploratory mutational analysis. The K716R mutation attenuated the CAG-associated increase in ubiquitin-associated EGFR signaling as well as several changes in lysosomal trafficking, protein turnover, MAPK signaling, and autophagic flux, indicating that alteration of this residue can influence these CAG-associated responses. However, these findings do not establish K716 as a direct, primary, or unique ubiquitin conjugation site because lysine-to-arginine substitution may also influence receptor conformation, stability, protein interactions, or the accessibility of other ubiquitination sites.

In summary, our findings indicate that CAG attenuates Ang II-induced cardiac remodeling and is associated with enhanced EGFR lysosomal turnover, accompanied by reduced MAPK phosphorylation, changes in autophagy-related processes, and attenuation of cardiac fibroblast activation. This process was associated with reduced EGFR accumulation; decreased phosphorylation of ERK, JNK, and p38; and reduced downstream MAPK-responsive reporter activities, accompanied by decreased fibrosis, inflammation, and oxidative stress, as well as improved autophagic flux. The pharmacological inhibition and CHX-chase experiments further clarified the regulation of EGFR turnover by CAG. BafA1 markedly reversed the CAG-induced reduction in EGFR protein abundance, whereas MG132 showed no significant restorative effect, indicating that EGFR reduction predominantly involved the lysosomal rather than the proteasomal pathway. Consistently, CHX-chase analysis showed that CAG accelerated the time-dependent loss of EGFR, whereas the K716R mutation delayed EGFR turnover. Together with the increased EGFR–LAMP1 colocalization, these findings support enhanced lysosome-associated EGFR turnover following CAG treatment. The attenuation of these changes by K716R further suggests that K716 is functionally involved in the regulation of EGFR stability and trafficking. However, the molecular basis underlying the CAG-associated increase in ubiquitin-associated EGFR signal remains unresolved. Because the present assay used pan-ubiquitin immunoprecipitation followed by EGFR immunoblotting, it cannot by itself distinguish covalent EGFR ubiquitination from co-immunoprecipitation of EGFR associated with other ubiquitinated proteins. The present study did not identify the responsible E3 ubiquitin ligase or determine whether CAG alters covalent EGFR ubiquitination through E3 ligase regulation, altered deubiquitination, or an indirect mechanism. Moreover, K716R attenuated the CAG-associated changes in EGFR turnover, MAPK phosphorylation, downstream MAPK-responsive reporter activities, autophagic flux, and fibroblast activation, supporting a functional relationship between EGFR turnover and these downstream cellular responses. However, these findings should not be interpreted as direct evidence that K716 itself is covalently ubiquitinated. Substitution of lysine with arginine may also influence local protein conformation, receptor stability, protein–protein interactions, or the accessibility of other ubiquitination sites. Therefore, K716 should be considered a functionally relevant residue rather than a confirmed primary, unique, or direct ubiquitin conjugation site. In addition, ERK, JNK, and p38 phosphorylation was assessed only at the terminal in vivo endpoint and at 24 h in vitro. Therefore, the temporal dynamics of MAPK activation and the time-dependent effects of CAG remain to be further investigated. Finally, it should be noted that although the K716R mutant attenuated several CAG-associated effects, EGFR knockdown or knockout was not performed; therefore, whether the protective effects of CAG are strictly dependent on EGFR remains to be established.

Despite these limitations, the present study provides several potential translational implications. CAG improved cardiac function and reduced myocardial fibrosis, inflammation, oxidative stress, and apoptosis in an Ang II-induced remodeling model, while also suppressing cardiac fibroblast activation in vitro. These findings highlight EGFR lysosomal turnover, together with changes in ubiquitin-associated EGFR signaling as a potentially modifiable process in pathological cardiac remodeling and provide a basis for further evaluating CAG as a candidate anti-remodeling compound. In addition, circulating EGFR-related changes may have potential value for disease stratification or pharmacodynamic monitoring, although this possibility requires validation in larger and independent clinical cohorts. Available pharmacokinetic evidence indicates that CAG is orally absorbed, but whether the 25–75 μM concentrations used in vitro are achievable in plasma or cardiac tissue remains unclear. Therefore, further studies should define its pharmacokinetics, bioavailability, dose–exposure relationship, cardiac tissue exposure, and long-term safety before clinical translation. Validation in adult human cardiac fibroblasts and comparison with established anti-remodeling therapies will also be necessary to determine its clinical applicability.

While these findings provide mechanistic insights into the actions of CAG, several limitations should be acknowledged. First, the acute murine model may not fully recapitulate the chronic and progressive nature of human heart failure. Second, MAPK phosphorylation was assessed only at selected endpoints, limiting characterization of its temporal dynamics and time-dependent responses to CAG. Third, the clinical cohort was relatively small and cross-sectional, and an independent serum validation cohort was unavailable. In addition, circulating soluble EGFR and myocardial EGFR transcript levels represent different biological layers. Therefore, the clinical significance of circulating EGFR requires further validation in larger, independent cohorts. Fourth, the potential contribution of EGFR ectodomain shedding or extracellular release to circulating EGFR levels was not directly examined. Fifth, autophagic flux was not directly assessed in vivo; therefore, the animal data reflect autophagy-related molecular and structural changes, while flux restoration was supported mainly by the tandem mRFP-GFP-LC3 assay in cardiac fibroblasts. Sixth, pan-ubiquitin immunoprecipitation followed by EGFR immunoblotting demonstrated changes in the ubiquitin-associated EGFR signaling but cannot distinguish covalent EGFR ubiquitination from co-immunoprecipitation of EGFR associated with other ubiquitinated proteins. K716 was evaluated as only one literature-guided candidate residue, and other potential lysine residues were not systematically screened. The K716R experiments therefore demonstrate only that alteration of this residue modifies several CAG-associated responses; they do not establish K716 as a direct, predominant, unique, or required ubiquitin conjugation site. The relevant E3 ubiquitin ligase remains unidentified, and whether CAG alters covalent EGFR ubiquitination through E3 ligase regulation, altered deubiquitination, or an indirect mechanism remains unresolved. Seventh, the present study did not selectively block EGFR degradation to determine whether the protective effects of CAG were attenuated or abolished, nor did it independently enhance EGFR degradation to determine whether such an intervention alone could reproduce the protective phenotype. In addition, plasma and cardiac tissue exposure to CAG was not measured, and the in vitro concentrations used in this study have not been demonstrated to be pharmacologically achievable in vivo. Taken together, our findings suggest that CAG attenuates Ang II-induced cardiac remodeling in association with enhanced EGFR lysosomal turnover, accompanied by changes in MAPK signaling, autophagy-related processes, and cardiac fibroblast phenotypes. The results support a functional role for K716 in CAG-associated EGFR regulation but do not establish K716 as a direct ubiquitination site or EGFR as a confirmed direct molecular target of CAG. Further studies using chronic models, direct target-engagement assays, site-specific ubiquitination analysis, and larger prospective clinical cohorts are required to validate these findings.

## 4. Materials and Methods

### 4.1. Reagents

CAG was obtained from Shanghai Yuanshi Technology Co., Ltd. (Shanghai, China). Angiotensin II (Ang II) was purchased from Shanghai Weihuan Biotechnology Co., Ltd. (Shanghai, China). Mouse EGFR ELISA Kit, IL-1β/IL-6/TNF-α ELISA kits, NT-proBNP ELISA kits, cTnI ELISA kits, CKMB ELISA kits, and CCK-8 kits were obtained from Shanghai Enzyme-linked Biotechnology Co., Ltd. (Shanghai, China). The human soluble EGFR SimpleStep ELISA Kit was obtained from Abcam (Cambridge, UK). HE, Masson, Sirius Red, EdU, crystal violet, ghost pen ring (phalloidin), and MDC staining kits were provided by Solarbio Science & Technology Co., Ltd. (Beijing, China). ROS staining kits and WGA staining kits were purchased from Sigma-Aldrich (St. Louis, MO, USA) and TUNEL kits were purchased from Solarbio Science & Technology Co., Ltd. (Beijing, China). Antibodies against EGFR (for Western blot and immunofluorescence) and LAMP1 were purchased from Santa Cruz Biotechnology (Dallas, TX, USA). Antibodies against IL-1β, IL-6, TNF-α, LC3A/B, Collagen I, Collagen III, Vimentin, and GAPDH were provided by Wuhan Sanying Biotechnology Co., Ltd. (Wuhan, China). PCR primers were synthesized by Shanghai Shenggong Bioengineering Co., Ltd. (Shanghai, China). Reverse transcription kits and SYBR Green qPCR kits were purchased from Nanjing Vazyme Biotech Co., Ltd. (Nanjing, China). Antibodies against ERK, p-ERK, JNK, p-JNK, P38, p-P38, LC3, Beclin-1, and P62 were supplied by Jiangsu Qingke Biological Research Center Co., Ltd. (Taizhou, China). The autophagy flux detection kit and dual-luciferase reporter assay kits were obtained from Shanghai Beyotime Biotechnology Co., Ltd. (Shanghai, China). EGFR K716R mutant plasmids were purchased from Nanjing Zebrafish Biotechnology Co., Ltd. (Nanjing, China).

### 4.2. Peripheral Blood Collection

Peripheral venous blood samples (5–10 mL) were collected from the antecubital vein of each participant under fasting conditions using standard aseptic procedures. Samples were drawn into EDTA-coated tubes and immediately centrifuged at 3000 rpm for 10 min at 4 °C to isolate plasma or serum, which was then aliquoted and stored at −80 °C until further analysis. All procedures involving human participants were approved by the Ethics Committee of Dongzhimen Hospital, Beijing University of Chinese Medicine (approval no. 2023DZMEC-228-03). Written informed consent was obtained from all participants in accordance with the ethical standards of the institutional and national research committees and the 1964 Declaration of Helsinki and its later amendments.

### 4.3. Animals

Male C57BL/6 mice (6 weeks old, 16–20 g, SPF grade) were obtained from Liaoning Changsheng Biotechnology Co., Ltd. (Shenyang, China, certificate no: SCXK (Liao): 2023–0001). All animals were housed in the Animal Center of Liaoning University of Traditional Chinese Medicine under standard conditions, with controlled temperature (20–25 °C) and humidity and a 12 h light/dark cycle. Food and water were provided ad libitum throughout the experimental period. All procedures were performed in accordance with the guidelines of the Institutional Animal Care and Use Committee (IACUC) of Liaoning University of Traditional Chinese Medicine. The experimental protocol was approved by the IACUC (approval no: 21000042023021, date: 3 March 2023).

### 4.4. Primary Cardiac Fibroblasts

Primary cardiac fibroblasts were isolated from 1–3-day-old neonatal C57BL/6 mice by enzymatically digesting minced heart tissue with 0.08% trypsin and 0.05% collagenase type II. The resulting cell suspension was filtered and pre-plated for 90 min in DMEM supplemented with 10% fetal bovine serum and 1% penicillin–streptomycin to allow selective adherence of fibroblasts. After reaching 70–80% confluence, the cells were treated with Ang II (100 μM) alone or in combination with CAG at the indicated concentrations for 24 h. Cells were subsequently collected for Western blotting, RT-qPCR, dual-luciferase reporter assays, and other indicated analyses. For experiments involving EGFR-WT or EGFR-K716R, cells were transfected with the corresponding plasmids for 24 h before Ang II and CAG treatment.

### 4.5. In Vivo Study

Male mice were randomly assigned to five groups: control, model, Ang II + Enalapril (10 mg/kg/day), Ang II + low-dose CAG (CAG-L, 100 mg/kg/day), and Ang II + high-dose CAG (CAG-H, 200 mg/kg/day) [48]. After a 7-day acclimatization period, all mice were implanted subcutaneously with Alzet osmotic minipumps (model 2004) on day 8, delivering either Ang II (500 ng/kg/min) [49] or saline for 28 consecutive days. Mice in the enalapril, CAG-L, and CAG-H groups received daily oral gavage of the corresponding treatments, starting from day 1 after minipump implantation, while the control and model groups received an equivalent volume of saline. After 28 days of treatment, transthoracic echocardiography was performed to assess cardiac function. Mice were then anesthetized for blood collection, and hearts were rapidly excised, photographed, and weighed. A portion of the heart tissue was fixed in 4% paraformaldehyde for histological analysis, while the remaining tissue was snap-frozen and stored at −80 °C for subsequent biochemical and molecular assays.

### 4.6. Clinical Biochemistry

The levels and activity of EGFR, NT-proBNP, CK-MB, cTnI, IL-1β, IL-6, and TNF-α in mouse serum were measured using commercial ELISA kits. All procedures were carried out in strict accordance with the manufacturers’ protocols. Serum samples were thawed on ice, diluted appropriately, and incubated in pre-coated 96-well plates. Absorbance was measured at the recommended wavelength using a microplate reader, and concentrations were calculated based on standard curves.

### 4.7. Hematoxylin and Eosin Staining

Hematoxylin and eosin (HE) staining was performed to evaluate myocardial histopathology. Briefly, heart tissues were fixed in 4% paraformaldehyde, embedded in paraffin, and sectioned at 5 μm thickness. The sections were deparaffinized, rehydrated, stained with hematoxylin and eosin, dehydrated, and mounted. Pathological changes such as myocardial necrosis, inflammatory cell infiltration, and myofiber disarray were examined under a light microscope.

### 4.8. Masson Staining

Masson staining was used to assess myocardial fibrosis. Heart tissues were fixed in 4% paraformaldehyde, embedded in paraffin, and sectioned at 5 μm thickness. Sections were deparaffinized, rehydrated, and stained using a Masson staining kit according to the manufacturer’s instructions. Collagen fibers were stained blue, muscle fibers red, and nuclei black.

### 4.9. Sirius Red Staining

Sirius Red staining was used to assess collagen deposition in myocardial tissue. Heart samples were fixed in 4% paraformaldehyde, embedded in paraffin, and cut into 5 μm sections. After deparaffinization and rehydration, the sections were stained using a Sirius Red staining kit, following the manufacturer’s instructions. Collagen fibers were stained red, allowing visualization and evaluation of myocardial fibrosis under a light microscope.

### 4.10. Reactive Oxygen Species Fluorescence Staining

Reactive oxygen species (ROS) levels in myocardial tissue were detected using an ROS fluorescence staining kit. Freshly collected heart tissues were embedded in OCT compound and cryosectioned at a thickness of 8 μm. Sections were incubated with DHE (dihydroethidium) working solution at 37 °C for 30 min in the dark. After rinsing with PBS, sections were observed under a fluorescence microscope. ROS generation was visualized as red fluorescence, and signal intensity was used to semi-quantitatively assess oxidative stress levels.

### 4.11. Terminal Deoxynucleotidyl Transferase dUTP Nick End Labeling Assay

Terminal deoxynucleotidyl transferase dUTP nick end labeling (TUNEL) staining was performed to evaluate cardiomyocyte apoptosis. Heart tissues were fixed in 4% paraformaldehyde, embedded in paraffin, and sectioned at a thickness of 5 μm. Sections were deparaffinized, rehydrated, and incubated with proteinase K at room temperature. After washing with PBS, sections were treated with the TUNEL reaction mixture according to the manufacturer’s instructions. Nuclei were counterstained with DAPI. Apoptotic nuclei were visualized as green fluorescence under a fluorescence microscope, and the proportion of TUNEL-positive cells was calculated to assess the degree of apoptosis.

### 4.12. Wheat Germ Agglutinin Staining

Wheat germ agglutinin (WGA) staining was used to assess cardiomyocyte cross-sectional area. Heart tissues were fixed in 4% paraformaldehyde, embedded in paraffin, and sectioned at 5 μm thickness. Sections were deparaffinized, rehydrated, and incubated with WGA conjugated to a fluorescent dye for membrane labeling. Nuclei were counterstained with DAPI. Images were captured using a fluorescence microscope, and cardiomyocyte area was quantified using ImageJ software (version 1.54, National Institutes of Health, Bethesda, MD, USA) to evaluate hypertrophic changes.

### 4.13. Immunohistochemistry

Immunohistochemistry (IHC) was performed to detect the expression of inflammatory markers in myocardial tissue. Heart samples were fixed in 4% paraformaldehyde, embedded in paraffin, and sectioned at 5 μm thickness. After deparaffinization and rehydration, sections underwent antigen retrieval, followed by incubation with primary antibodies against IL-1β, IL-6, or TNF-α. After washing, sections were incubated with HRP-conjugated secondary antibodies and developed with DAB chromogen. Hematoxylin was used for nuclear counterstaining. Positive staining appeared brown under light microscopy and was quantified using ImageJ software.

### 4.14. Immunofluorescence Staining

Immunofluorescence (IF) staining was performed on 5 μm thick paraffin-embedded heart tissue sections. After deparaffinization and rehydration, antigen retrieval was carried out, followed by blocking with 5% BSA for 1 h at room temperature. Sections were then incubated overnight at 4 °C with primary antibodies. After washing, appropriate fluorescently labeled secondary antibodies were applied for 1 h at room temperature in the dark. Nuclei were counterstained with DAPI. Images were captured using a fluorescence microscope, and signal intensity was analyzed using ImageJ software.

### 4.15. Transmission Electron Microscopy

Transmission electron microscopy (TEM) was used to observe ultrastructural features of cardiomyocytes. Heart tissue samples were fixed in 2.5% glutaraldehyde at 4 °C, post-fixed in osmium tetroxide, dehydrated through a graded ethanol series, and embedded in epoxy resin. Ultrathin sections (70–90 nm) were cut using an ultramicrotome, stained with uranyl acetate and lead citrate, and examined under a transmission electron microscope to assess mitochondrial morphology and autophagosome formation.

### 4.16. 5-Ethynyl-2′-Deoxyuridine Incorporation Assay

5-ethynyl-2′-deoxyuridine (EdU) staining was used to evaluate cell proliferation. Cells were seeded into 24-well plates and incubated with EdU working solution for 2 h at 37 °C. After incubation, cells were fixed with 4% paraformaldehyde, permeabilized with 0.5% Triton X-100, and reacted with the EdU detection cocktail according to standard procedures. Nuclei were counterstained with DAPI. Fluorescent signals were captured using a fluorescence microscope, and EdU-positive cells were quantified to assess DNA synthesis activity.

### 4.17. Scratch Wound Healing Assay

The wound healing assay was performed to assess cell migratory capacity. Fibroblasts were seeded into 6-well plates and cultured to near confluence. A straight scratch was made across the cell monolayer using a sterile 200 μL pipette tip. Detached cells were gently removed with PBS, and fresh, serum-free medium was added. Images of the scratch area were taken at 0 h and 24 h under a microscope. The wound closure area was quantified to evaluate cell migration.

### 4.18. Colony Formation Assay

For the colony formation assay, fibroblasts were seeded at low density into 6-well plates and cultured in complete medium for 10 days to allow colony development. At the endpoint, colonies were washed with PBS, fixed with 4% paraformaldehyde for 20 min, and stained with crystal violet for 15 min. The number of colonies containing at least 50 cells was counted under a microscope to assess clonogenic ability.

### 4.19. Phalloidin Staining

Phalloidin staining was performed to visualize F-actin structures in fibroblasts. After treatment, cells were fixed with 4% paraformaldehyde for 20 min and permeabilized with 0.1% Triton X-100 for 10 min at room temperature. Cells were then incubated with fluorescently labeled phalloidin for 30 min in the dark. Nuclei were counterstained with DAPI. F-actin distribution was observed using a fluorescence microscope, and fluorescence intensity was quantified to assess cytoskeletal remodeling.

### 4.20. Pan-Ubiquitin Immunoprecipitation Followed by EGFR Immunoblotting

Cell lysates were prepared on ice using RIPA lysis buffer (Thermo Fisher Scientific, Waltham, MA, USA, Cat. No. 89900) supplemented with a protease inhibitor cocktail (Thermo Fisher Scientific, Cat. No. 78430). N-ethylmaleimide (NEM; Sigma-Aldrich, cat. no. 04260) was added during cell lysis to minimize deubiquitination. After centrifugation, equal amounts of total protein were used for immunoprecipitation, and a portion of each lysate was retained as the input sample. The remaining lysates were incubated overnight at 4 °C with a pan-ubiquitin antibody (P4D1; Santa Cruz Biotechnology, cat. no. sc-8017). Species- and isotype-matched normal mouse IgG1 (Santa Cruz Biotechnology, cat. no. sc-3877) was used as the negative IP control. Pierce Protein A/G Magnetic Beads (Thermo Fisher Scientific, cat. no. 88802) were subsequently added and incubated for 2 h at 4 °C. The immune complexes were washed three times with lysis buffer and eluted by boiling in SDS sample buffer. The immunoprecipitated proteins were separated by SDS-PAGE, transferred onto PVDF membranes, and immunoblotted with an anti-EGFR antibody (A-10; Santa Cruz Biotechnology, cat. no. sc-373746). Signals were visualized by enhanced chemiluminescence and quantified by densitometry.

### 4.21. Monodansylcadaverine Staining

Cells were seeded onto glass coverslips and treated according to experimental protocols. Following treatment, cells were washed with PBS and incubated with monodansylcadaverine (MDC) working solution at 37 °C for 30 min in the dark. After incubation, cells were rinsed three times with PBS to remove excess dye and immediately imaged under a fluorescence microscope. MDC-positive autophagic vacuoles were visualized as punctate fluorescent signals and quantified using ImageJ software.

### 4.22. mRFP-GFP-LC3 Autophagic Flux Assay

Cells were transfected with the tandem mRFP-GFP-LC3 plasmid using Lipofectamine 3000 according to standard protocols. After 24 h of transfection, cells were subjected to the indicated treatments. Following incubation, cells were washed with PBS, fixed with 4% paraformaldehyde for 15 min at room temperature, and then rinsed again with PBS. Fluorescent images were captured using a confocal laser scanning microscope. Autophagosomes were identified as yellow puncta (mRFP + GFP+) and autolysosomes as red-only puncta (mRFP + GFP−), and the puncta were quantified using ImageJ software to assess autophagic flux.

### 4.23. EGFR K716R Point Mutation Plasmid Transfection

EGFR K716R mutant plasmids were constructed by site-directed mutagenesis, replacing lysine (K) at position 716 with arginine (R). The mutation was confirmed by DNA sequencing. Cells were transfected with either wild-type EGFR or EGFR-K716R plasmids using Lipofectamine 3000 according to the manufacturer’s instructions. After 24 h of transfection, cells were treated with Ang II and/or CAG as indicated.

### 4.24. Flow Cytometry for Membrane EGFR

Cells were harvested and washed twice with cold PBS, then resuspended in PBS containing 1% BSA to block nonspecific binding. To specifically assess membrane-bound EGFR, cells were incubated with a primary antibody against EGFR on ice for 30 min without permeabilization. After washing, cells were labeled with a fluorophore-conjugated secondary antibody for another 30 min in the dark on ice. Following final washes, flow cytometry was performed. During analysis, doublets were excluded using forward scatter height (FSC-H) versus forward scatter area (FSC-A), and live single cells were gated based on side scatter (SSC) and FSC profiles. Fluorescence intensity within the gated population was used to quantify surface EGFR expression.

### 4.25. EGFR–LAMP1 Colocalization by IF

Immunofluorescence staining was used to assess the intracellular colocalization of EGFR and LAMP1. Cells were fixed with 4% paraformaldehyde, permeabilized with 0.3% Triton X-100, and blocked with 5% bovine serum albumin (BSA). Cells were then incubated with primary antibodies against EGFR and LAMP1 overnight at 4 °C, followed by fluorescent secondary antibodies conjugated with Alexa Fluor 488 (green) and Alexa Fluor 594 (red), respectively. Nuclei were counterstained with DAPI. Stained cells were visualized using a confocal laser scanning microscope. Colocalization was analyzed by evaluating the degree of fluorescence overlap between EGFR and LAMP1 signals.

### 4.26. Dual-Luciferase Reporter Assay

Dual-luciferase reporter assays were performed to assess downstream transcriptional responses associated with ERK, JNK, and p38 signaling. Cells were seeded in 24-well plates and co-transfected with the pFR-Luc firefly luciferase reporter plasmid and the corresponding pathway-specific transactivator plasmid, including pFA2-Elk1 for ERK-associated signaling, pFA2-c-Jun for JNK-associated signaling, or pFA-CHOP for p38-associated signaling (Table 2). The pRL-TK plasmid was co-transfected as a Renilla luciferase internal control. Where indicated, EGFR-WT or EGFR-K716R expression plasmids were also introduced into the cells. Transfection was performed using Lipofectamine 3000 according to the manufacturer’s instructions. After 24 h, the cells were subjected to the indicated treatments. Firefly and Renilla luciferase activities were sequentially measured using a dual-luciferase reporter assay system and a microplate luminometer. Firefly luciferase activity was normalized to Renilla luciferase activity. The normalized values were interpreted as ERK/Elk-1-, JNK/c-Jun-, and p38/CHOP-responsive reporter activities, respectively, rather than the transcriptional activities of ERK, JNK, or p38 themselves.

### 4.27. Pharmacological Inhibition of EGFR Degradation

To distinguish lysosomal degradation from proteasomal degradation, the cells were divided into four groups: Ang II, Ang II + CAG, Ang II + CAG + MG132, and Ang II + CAG + bafilomycin A1 (BafA1). MG132 (10 μM) or BafA1 (100 nM) was added during the final 4 h before cell collection. An equivalent volume of dimethyl sulfoxide was added to the corresponding vehicle-control groups. Following treatment, the cells were washed twice with ice-cold phosphate-buffered saline and lysed using RIPA buffer supplemented with protease and phosphatase inhibitors. EGFR and β-actin protein levels were determined by Western blotting. Band intensities were quantified using ImageJ software. EGFR protein abundance was normalized to the corresponding β-actin signal.

### 4.28. Cycloheximide-Chase Assay

Primary cardiac fibroblasts were divided into the following groups: Ang II, Ang II + CAG, Ang II + CAG + EGFR-WT, and Ang II + CAG + EGFR-K716R. Following the indicated treatments, the culture medium was replaced with fresh complete medium containing cycloheximide (CHX) at a final concentration of 50 μg/mL to inhibit de novo protein synthesis. Cells were harvested at 0, 6, 12, and 24 h after CHX administration. At each time point, cells were washed twice with ice-cold phosphate-buffered saline and lysed using RIPA buffer supplemented with protease and phosphatase inhibitors. EGFR and β-actin protein levels were determined by Western blotting.

### 4.29. Public Transcriptomic Dataset Analysis

The publicly available RNA-sequencing dataset GSE161472 was obtained from the Gene Expression Omnibus (GEO) database. This dataset contains transcriptomic profiles from four cardiac chambers of non-failing (NF) donors and patients with heart failure with reduced ejection fraction (HFrEF). For the present analysis, only left ventricular samples were included, comprising 9 NF and 12 HFrEF samples. EGFR expression data were extracted from the processed expression matrix according to the corresponding sample annotations. Receiver operating characteristic (ROC) curve analysis was performed using EGFR expression as the test variable and HFrEF status as the classification variable. The discriminatory performance was evaluated by calculating the area under the ROC curve (AUC).

### 4.30. RT-qPCR Analysis

Total RNA was extracted from myocardial tissues and cultured fibroblasts using a standard phenol–chloroform method, and RNA concentration and purity were assessed spectrophotometrically. Reverse transcription was subsequently performed to synthesize cDNA, followed by quantitative PCR amplification using SYBR Green-based detection. Gene-specific primers were designed for Collagen I, Collagen III, and Vimentin, with GAPDH serving as the internal control (Table 3). Relative gene expression levels were calculated using the 2^−ΔΔCt^ method.

### 4.31. Western Blotting

Western blotting was used to assess protein expression in myocardial tissue and cultured fibroblasts. Heart tissues or cells were lysed using RIPA buffer supplemented with protease and phosphatase inhibitors. Protein concentrations were determined using a BCA assay. Equal amounts of protein were separated by SDS-PAGE and transferred to PVDF membranes. Membranes were blocked with 5% non-fat milk and incubated overnight at 4 °C with primary antibodies against target proteins. After washing, membranes were incubated with appropriate HRP-conjugated secondary antibodies. Protein bands were visualized using enhanced chemiluminescence and quantified using densitometry analysis. GAPDH was used as the loading control.

### 4.32. Statistical Analysis

Statistical analyses were performed using GraphPad Prism 9.0, and data are presented as the mean ± SD. Normality and variance homogeneity were assessed using the Shapiro–Wilk and Brown–Forsythe tests, respectively. Normally distributed data were analyzed using one-way ANOVA with Bonferroni’s post hoc test or Welch’s ANOVA with Dunnett’s T3 test when variances were unequal. Non-normally distributed data were analyzed using the Kruskal–Wallis test with Dunn’s post hoc test. CHX time-course data were analyzed using two-way ANOVA with Bonferroni correction. Sample size was estimated using G*Power 3.1.9.7 with α = 0.05 and 80% power. All tests were two-sided, and *p* < 0.05 was considered statistically significant.

## 5. Conclusions

This study suggests that EGFR may serve as a potential molecular contributor to adverse cardiac remodeling. CAG alleviated structural and functional deterioration in a murine model of Ang II-induced cardiac stress. CAG increased the EGFR signal recovered in pan-ubiquitin immunoprecipitates and was associated with enhanced lysosome-associated EGFR turnover. These changes occurred in parallel with reduced MAPK phosphorylation, attenuation of fibrotic and inflammatory responses, and changes in autophagy-related processes. The K716R mutation attenuated CAG-associated changes in EGFR turnover, MAPK signaling, autophagy-related processes, and fibroblast phenotypes, indicating that alteration of K716 modifies several CAG-associated responses. However, the present findings do not directly establish K716 as a covalent ubiquitin conjugation site. Importantly, these findings do not establish EGFR degradation as either necessary or sufficient for the cardioprotective effects of CAG. In addition, untargeted metabolomics revealed that CAG-regulated metabolites were enriched in pathways related to ErbB and MAPK signaling, suggesting potential systemic effects. Nevertheless, these observations were obtained from a single preclinical model with a relatively short intervention period, and the causal relationships between metabolic alterations and cardiac outcomes require further investigation. Collectively, these findings suggest that CAG attenuates Ang II-induced cardiac remodeling in association with enhanced EGFR lysosomal turnover, accompanied by changes in MAPK signaling, autophagy-related processes, and fibroblast phenotypes. These findings support a functional association among these responses rather than an established linear causal pathway. Further long-term mechanistic and clinical studies are required to clarify the causal relationships among these processes and establish the therapeutic relevance of CAG.

## Figures and Tables

**Table 1:** Baseline clinical characteristics of patients across NYHA functional classes (I–IV).

	NYHA I *N* = 12	NYHA II *N* = 12	NYHA III *N* = 12	NYHA IV *N* = 12	*P/H/χ* ^2^
Age	62.50 ± 9.82	59.92 ± 12.60	60.42 ± 6.16	59.58 ± 10.12	0.908/0.55
Sex					
Male	7 (58.3%)	6 (50.0%)	3 (25.0%)	4 (33.3%)	0.330/3.43
Female	5 (41.7%)	6 (50.0%)	9 (75.0%)	8 (66.7%)	
Smoking History					
Male	10 (83.3%)	8 (66.7%)	9 (75.0%)	9 (75.0%)	0.828/0.89
Female	2 (16.7%)	4 (33.3%)	3 (25.0%)	3 (25.0%)	
Alcohol Consumption					
Male	12 (100%)	11 (91.7%)	8 (66.7%)	10 (83.3%)	0.119/5.85
Female	0 (0%)	1 (8.3%)	4 (33.3%)	2 (16.7%)	
Medical History					0.572/2.00
Male	6 (50.0%)	7 (58.30%)	7 (58.30%)	4 (33.3%)	
Female	6 (50.0%)	5 (41.70%)	5 (41.70%)	8 (66.7%)	
History of Diabetes Mellitus					0.330/3.43
Male	5 (41.7%)	8 (66.7%)	9 (75.0%)	6 (50.0%)	
Female	7 (58.3%)	4 (33.3%)	3 (25.0%)	6 (50.0%)	
Medications					
ACEI/ARB Usage					0.112/6.00
Male	4 (33.3%)	9 (75.0%)	4 (33.3%)	7 (58.30%)	
Female	8 (66.7%)	3 (25.0%)	8 (66.7%)	5 (41.70%)	
Antiplatelet Therapy					0.753/1.20
Male	1 (8.3%)	2 (16.7%)	2 (16.7%)	3 (25.0%)	
Female	11 (91.7%)	10 (83.3%)	10 (83.3%)	9 (75.0%)	

Values are presented as mean ± standard deviation (SD) for continuous variables and *n* (%) for categorical variables. Statistical comparisons were performed using one-way ANOVA (H test) or Chi-square test (*χ*^2^), as appropriate.

**Table 2:** Common MAPK pathway-responsive dual-luciferase reporter systems.

MAPK Branch	Actual Transactivator Plasmid	Reporter Plasmid	Readout
ERK	pFA2-Elk1	pFR-Luc	ERK/Elk-1-responsive reporter activity
JNK	pFA2-c-Jun	pFR-Luc	JNK/c-Jun-responsive reporter activity
p38	pFA-CHOP	pFR-Luc	p38/CHOP-responsive reporter activity

**Table 3:** RNA sequences.

mRNA	Primer Sequence (5′–3′)	
Collagen I	Forward:	GGAACCTTCGCTTCCATACTC
	Reverse:	ACTGGTACATCAGCCCAAAC
Collagen III	Forward:	GCCATCCTCTAGAACTGTGTAAG
	Reverse:	CAGGCCAATGGCAATGTAAG
Vimentin	Forward:	CGGCTGCGAGAGAAATTGC
	Reverse:	CCACTTTCCGTTCAAGGTCAAG
GAPDH	Forward:	TCTCTGCTCCTCCCTGTTCT
	Reverse:	TACGGCCAAATCCGTTCACA

## Data Availability

The original contributions presented in this study are included in the article/Supplementary Materials. Further inquiries can be directed to the corresponding authors.

## Supplementary Materials

The following supporting information can be downloaded at https://www.mdpi.com/article/10.3390/ph19091360/s1: Figure S1. (A) Representative images of ROS fluorescence staining (red), with nuclei counterstained by DAPI (blue); merged images shown below. (B) TUNEL staining (green) indicating apoptotic cardiomyocytes, with nuclei stained by DAPI (blue). (C) WGA staining (green) outlining cell membranes for analysis of cardiomyocyte cross-sectional area; nuclei stained by DAPI (blue). (D–F) Quantification of ROS fluorescence intensity, percentage of TUNEL-positive nuclei, and cardiomyocyte cross-sectional area. Data are presented as mean ± SD (*n* = 5). ** *p* < 0.01 vs. control group; # *p* < 0.05, ## *p* < 0.01 vs. model group, ns, not significant; Figure S2. (A) Immunofluorescence staining of EGFR (green) and nuclei (DAPI, blue). (B) Representative images of LC3A (green), LC3B (red), and DAPI (blue) immunofluorescence staining. (C) EdU staining and quantification of EdU-positive cells. (D) Wound healing assay at 0 h and 24 h, with quantification of recovered area. (E) Colony formation assay and colony number analysis. (F) Representative immunofluorescence images of phalloidin staining (green) showing F-actin distribution; nuclei were counterstained with DAPI (blue). (G) Immunofluorescence staining for EGFR (green) and nuclei (DAPI, blue) in fibroblasts. (H) Representative images of MDC staining for autophagic vacuoles. (I) Representative images from the mRFP-GFP-LC3 assay showing autophagosomes (yellow: merged GFP + RFP) and autolysosomes (red only). Data are presented as mean ± SD (*n* = 5). ** *p* < 0.01 vs. control group; # *p* < 0.05, ## *p* < 0.01 vs. model group, ns, not significant; Figure S3. (A) Wound healing assay shows migration capacity in CAG, CAG-Vector, and CAG-EGFR716R groups after 24 h. (B) EdU incorporation assay quantifies proliferative cells (red) with DAPI nuclear counterstaining (blue). (C) Phalloidin staining visualizes F-actin filaments (green) with DAPI (blue). (D) Colony formation assay and quantification of colony number. (E) The EGFR expression levels in the serum of mice from each group were measured by ELISA. (F) ELISA showed that CAG reduced EGFR expression in the cell supernatant, while the 716R mutation reversed this effect. Data are presented as mean ± SD (*n* = 5). ** *p* < 0.01 vs. control group; ## *p* < 0.01 vs. model group, aap < 0.01 vs. CAG group, ns, not significant; Figure S4. CAG-associated EGFR turnover is predominantly lysosome-associated and attenuated by K716R. (A,B) Representative Western blots and quantification of EGFR in Ang II-stimulated cardiac fibroblasts treated with CAG, MG132, or BafA1. (C,D) Representative Western blots and quantification of EGFR remaining at 0, 6, 12, and 24 h after CHX treatment in the indicated groups. Values were normalized to β-actin and the corresponding 0 h value. (E) Apparent EGFR half-life estimated using a one-phase exponential decay model. Data are presented as mean ± SD (*n* = 3). (F) Forest plot showing the associations of circulating EGFR with LVEF, ln(BNP), LVD, LAD, IVS, and EDV in multivariable linear regression models adjusted for age and sex. Regression coefficients (B) and 95% confidence intervals are shown. (G) Independent exploratory ROC analysis of myocardial EGFR transcript expression for distinguishing HFrEF from non-failing left ventricular samples in the GSE161472 dataset. The AUC was 0.870. This analysis was not used as validation of circulating soluble EGFR. Panels B and E were analyzed by one-way ANOVA and panel D by two-way ANOVA, followed by Bonferroni’s multiple-comparisons test. * *p* < 0.05, ** *p* < 0.01, *** *p* < 0.001, **** *p* < 0.0001, ns, not significant.

## Abbreviations

ACEI	angiotensin-converting enzyme inhibitor
Ang II	angiotensin II
ARB	angiotensin receptor blocker
ASV	amplicon sequence variant
AUC	area under the curve
BNP	brain natriuretic peptide
CAG	cycloastragenol
ChIP	chromatin immunoprecipitation
CKMB	creatine kinase-MB
DAPI	4′,6-diamidino-2-phenylindole
DHE	dihydroethidium
DMEM	Dulbecco’s Modified Eagle Medium
EdU	5-ethynyl-2′-deoxyuridine
EF	ejection fraction
EGFR	epidermal growth factor receptor
ELISA	enzyme-linked immunosorbent assay
ERK	extracellular signal-regulated kinase
GAPDH	glyceraldehyde-3-phosphate dehydrogenase
HE	hematoxylin and eosin
HRP	horseradish peroxidase
IACUC	Institutional Animal Care and Use Committee
IF	immunofluorescence
IHC	immunohistochemistry
IL	interleukin
IP	immunoprecipitation
JNK	c-Jun N-terminal kinase
KEGG	Kyoto Encyclopedia of Genes and Genomes
LAMP1	lysosome-associated membrane protein 1
LC3	microtubule-associated protein 1A/1B-light chain 3
LVIDd	left ventricular internal diameter in diastole
LVIDs	left ventricular internal diameter in systole
LVFS	left ventricular fractional shortening
LVEF	left ventricular ejection fraction
MAPK	mitogen-activated protein kinase
MDC	monodansylcadaverine
NMDS	non-metric multidimensional scaling
NT-proBNP	N-terminal pro-brain natriuretic peptide
P62	sequestosome 1
PCA	principal component analysis
PCR	polymerase chain reaction
PERMANOVA	permutational multivariate analysis of variance
PLS-DA	partial least squares discriminant analysis
qPCR	quantitative polymerase chain reaction
ROS	reactive oxygen species
SDS-PAGE	sodium dodecyl sulfate–polyacrylamide gel electrophoresis
TUNEL	terminal deoxynucleotidyl transferase dUTP nick end labeling
UPLC	ultra-performance liquid chromatography
WB	Western blot
WGA	wheat germ agglutinin
