# Supplementary material for: Cycloastragenol Attenuates Angiotensin II-Induced Cardiac Remodeling in Association with Enhanced EGFR Lysosomal Turnover and Changes in MAPK Signaling and Autophagy
Source: Pharmaceuticals (Basel). 2026 Aug 27;19(9):1360. doi: 10.3390/ph19091360 (PMC13610723; doi:10.3390/ph19091360)
Supplement: Supplementary file 1 [file pharmaceuticals-19-01360-s001.zip › pharmaceuticals-4508099-supplementary.pdf]

Supplementary Materials

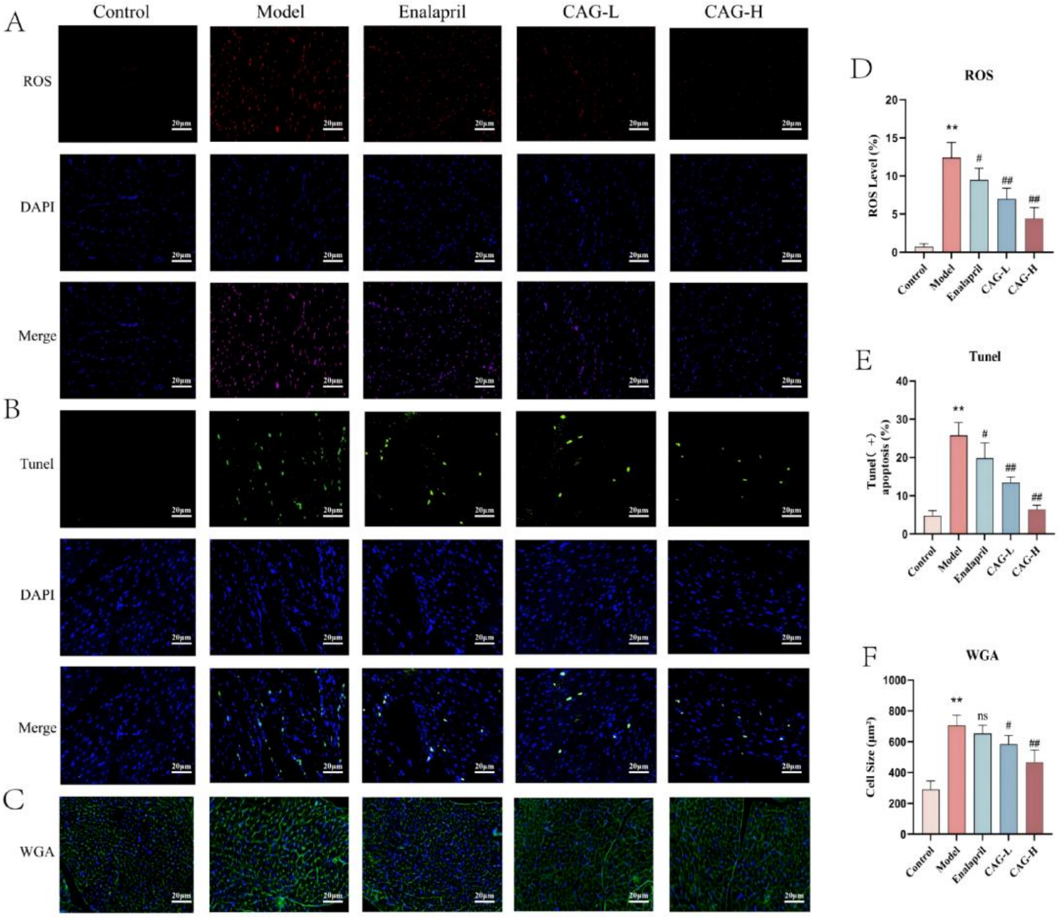


**Figure S1.** (**A**) Representative images of ROS fluorescence staining (red), with nuclei counterstained by DAPI (blue); merged images shown below. (**B**) TUNEL staining (green) indicating apoptotic cardiomyocytes, with nuclei stained by DAPI (blue). (**C**) WGA staining (green) outlining cell membranes for analysis of cardiomyocyte cross-sectional area; nuclei stained by DAPI (blue). (**D**–**F**) Quantification of ROS fluorescence intensity, percentage of TUNEL-positive nuclei, and cardiomyocyte cross-sectional area. Data are presented as mean ± SD (n = 5). ** $p < 0.01$ vs. control group; # $p < 0.05$, ## $p < 0.01$ vs. model group, ns, not significant.

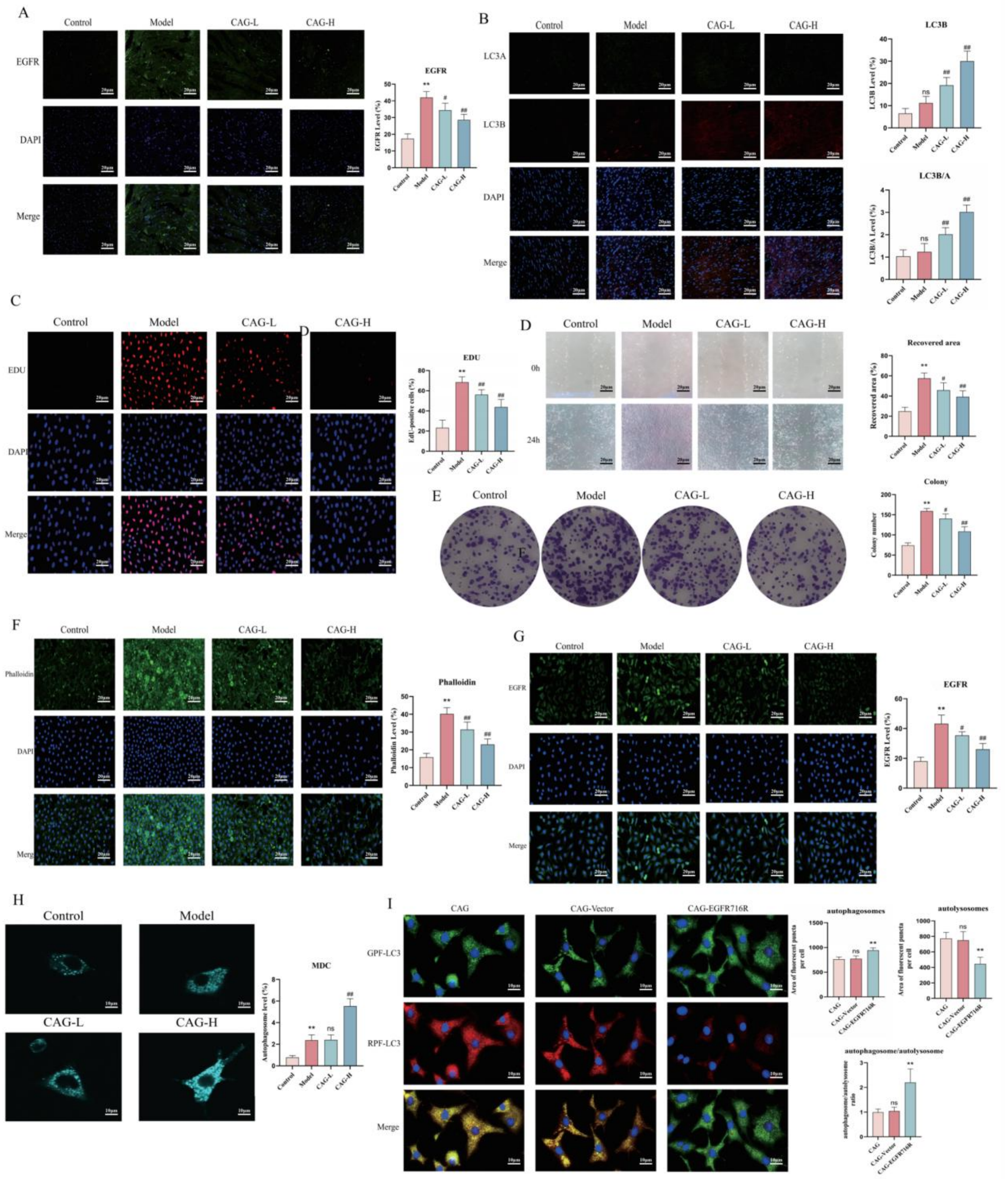


**Figure S2.** (**A**) Immunofluorescence staining of EGFR (green) and nuclei (DAPI, blue). (**B**) Representative images of LC3A (green), LC3B (red), and DAPI (blue) immunofluorescence staining. (**C**) EdU staining and quantification of EdU-positive cells. (**D**) Wound healing assay at 0 h and 24 h, with quantification of recovered area. (**E**) Colony formation assay and colony number analysis. (**F**) Representative immunofluorescence images of phalloidin staining (green) showing F-actin distribution; nuclei were counterstained with DAPI (blue). (**G**) Immunofluorescence staining for EGFR (green) and nuclei (DAPI, blue) in fibroblasts. (**H**) Representative images of MDC staining for autophagic vacuoles. (**I**) Representative images from the mRFP-GFP-LC3 assay showing autophagosomes (yellow: merged GFP + RFP) and autolysosomes (red only). Data are presented as mean ± SD

(n = 5). ** $p < 0.01$ vs. control group; # $p < 0.05$, ## $p < 0.01$ vs. model group, ns, not significant.

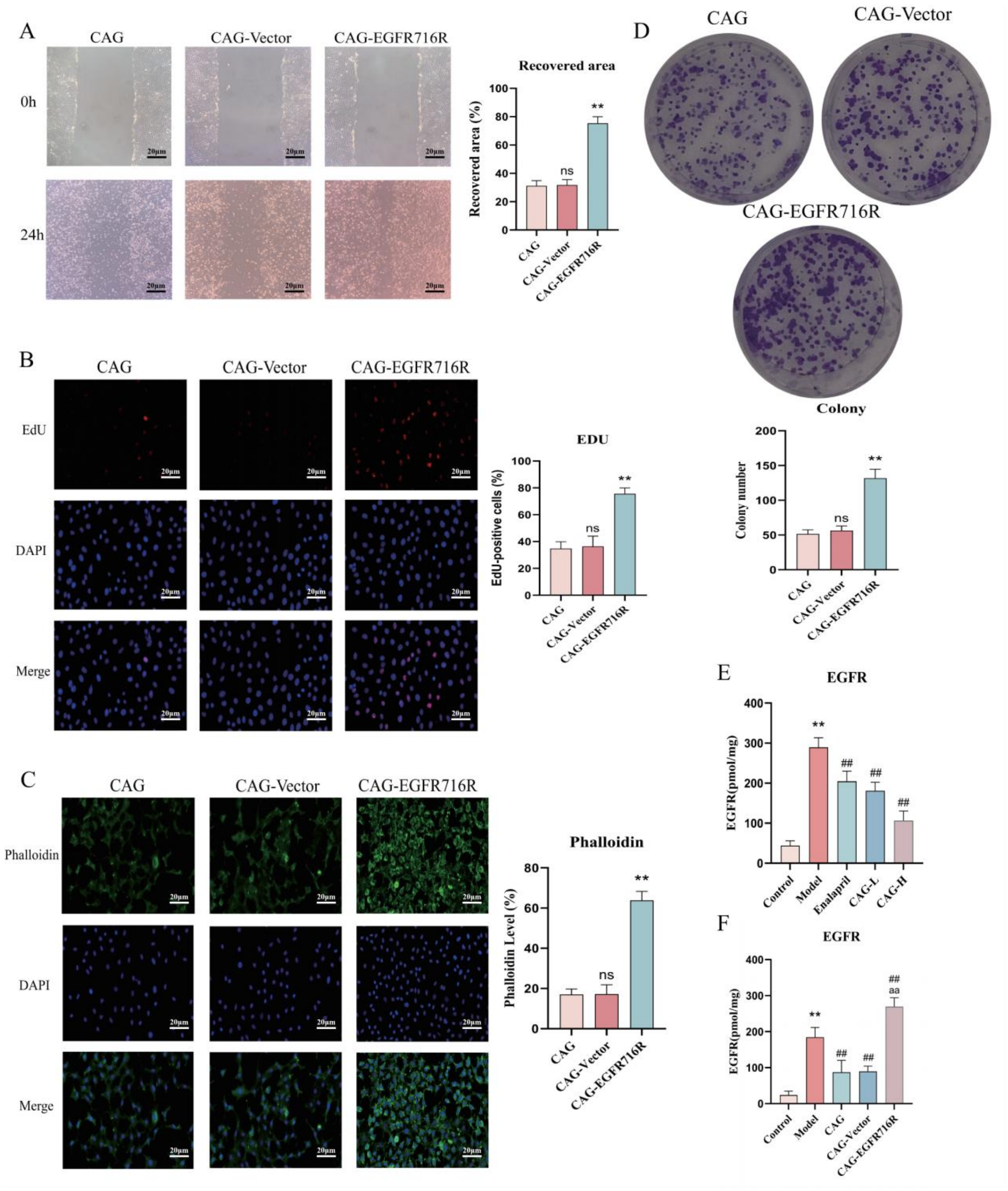


**Figure S3.** (**A**) Wound healing assay shows migration capacity in CAG, CAG-Vector, and CAG-EGFR716R groups after 24 h. (**B**) EdU incorporation assay quantifies proliferative cells (red) with DAPI nuclear counterstaining (blue). (**C**) Phalloidin staining visualizes F-actin filaments (green) with DAPI (blue). (**D**) Colony formation assay and quantification of colony number. (**E**) The EGFR expression levels in the serum of mice from each group were measured by ELISA. (**F**) ELISA showed that CAG reduced EGFR expression in the cell

supernatant, while the 716R mutation reversed this effect. Data are presented as mean ± SD (n = 5). ** $p < 0.01$ vs. control group; ## $p < 0.01$ vs. model group, aap < 0.01 vs. CAG group, ns, not significant.

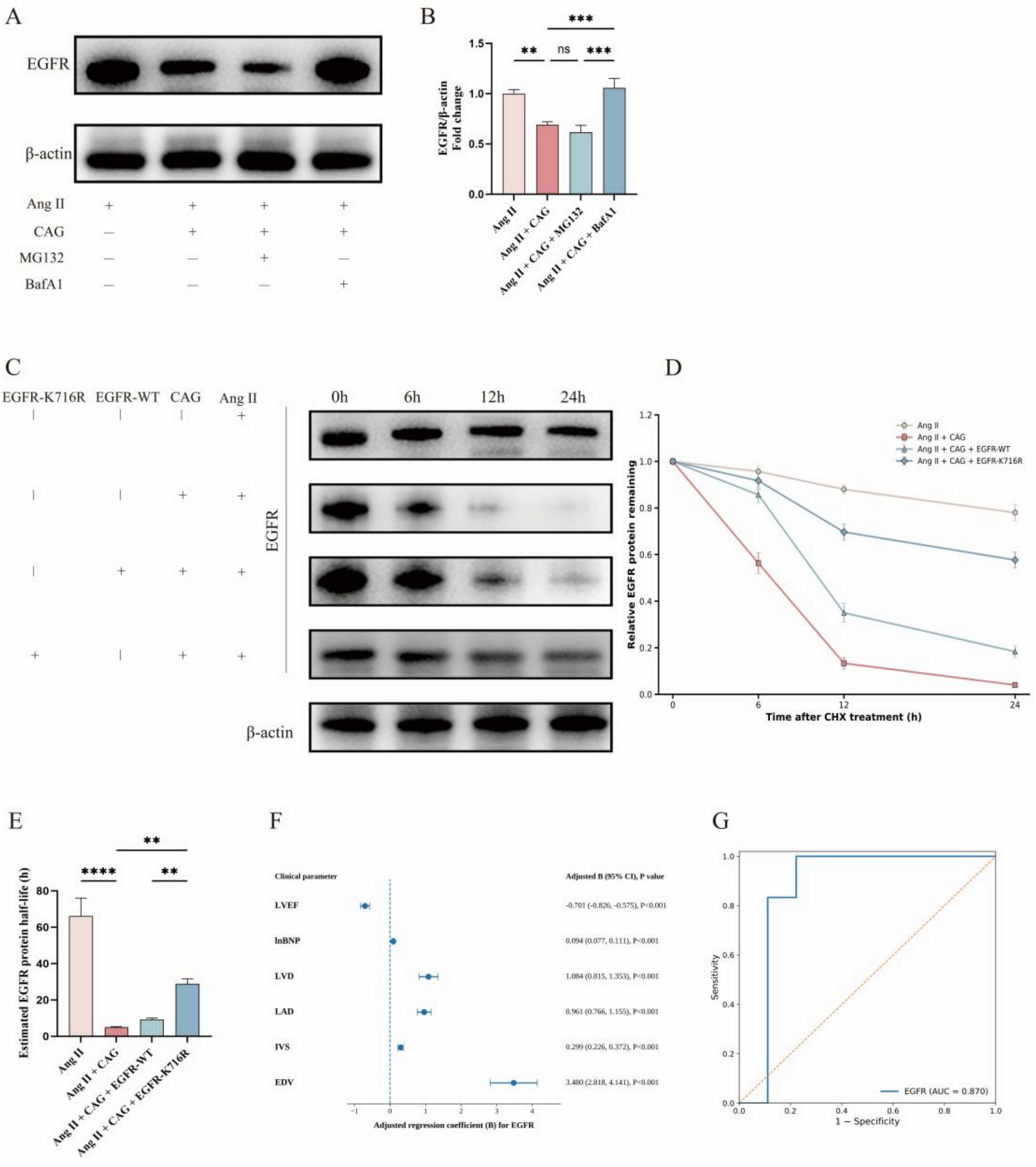


**Figure S4.** CAG-associated EGFR turnover is predominantly lysosome-associated and attenuated by K716R. (**A**,**B**) Representative Western blots and quantification of EGFR in Ang II-stimulated cardiac fibroblasts treated with CAG, MG132, or BafA1. (**C**,**D**) Representative Western blots and quantification of EGFR remaining at 0, 6, 12, and 24 h after CHX treatment in the indicated groups. Values were normalized to β-actin and the corresponding 0 h value. (**E**) Apparent EGFR half-life estimated using a one-phase exponential decay model.

Data are presented as mean ± SD (n = 3). (**F**) Forest plot showing the associations of circulating EGFR with LVEF, ln(BNP), LVD, LAD, IVS, and EDV in multivariable linear regression models adjusted for age and sex. Regression coefficients (**B**) and 95% confidence intervals are shown. (**G**) Independent exploratory ROC analysis of myocardial EGFR transcript expression for distinguishing HFrEF from non-failing left ventricular samples in the GSE161472 dataset. The AUC was 0.870. This analysis was not used as validation of circulating soluble EGFR. Panels B and E were analyzed by one-way ANOVA and panel D by two-way ANOVA, followed by Bonferroni's multiple-comparisons test. * $p < 0.05$, ** $p < 0.01$, *** $p < 0.001$, **** $p < 0.0001$, ns, not significant.
